# Glucose restriction enhances oxidative fiber formation: A multi-omic signal network involving AMPK and CaMK2

**DOI:** 10.1016/j.isci.2023.108590

**Published:** 2023-11-29

**Authors:** Kaiyi Zhang, Ning Xie, Huaqiong Ye, Jiakun Miao, Boce Xia, Yu Yang, Huanqi Peng, Shuang Xu, Tianwen Wu, Cong Tao, Jinxue Ruan, Yanfang Wang, Shulin Yang

**Affiliations:** 1 State Key Laboratory of Animal Biotech Breeding, Institute of Animal Science, Chinese Academy of Agricultural Sciences (CAAS), Beijing 100193, China; 2 Precision Livestock and Nutrition Unit, Gembloux Agro-Bio Tech, TERRA Teaching and Research Centre, Liège University, 5030 Gembloux, Belgium; 3 Key Laboratory of Agricultural Animal Genetics, Breeding and Reproduction of Ministry of Education & Key Lab of Swine Genetics and Breeding of Ministry of Agriculture and Rural Affairs, Huazhong Agricultural University, Wuhan 430070, China

**Keywords:** Comp, Metabolomics, Omics, Genomics, Proteomics

## Abstract

Skeletal muscle is a highly plastic organ that adapts to different metabolic states or functional demands. This study explored the impact of permanent glucose restriction (GR) on skeletal muscle composition and metabolism. Using *Glut4*^*m*^ mice with defective glucose transporter 4, we conducted multi-omics analyses at different ages and after low-intensity treadmill training. The oxidative fibers were significantly increased in *Glut4*^*m*^ muscles. Mechanistically, GR activated AMPK pathway, promoting mitochondrial function and beneficial myokine expression, and facilitated slow fiber formation via CaMK2 pathway. Phosphorylation-activated Perm1 may synergize AMPK and CaMK2 signaling. Besides, MAPK and CDK kinases were also implicated in skeletal muscle protein phosphorylation during GR response. This study provides a comprehensive signaling network demonstrating how GR influences muscle fiber types and metabolic patterns. These insights offer valuable data for understanding oxidative fiber formation mechanisms and identifying clinical targets for metabolic diseases.

## Highlights

• A multi-omic atlas illustrates skeletal muscle adaptation to glucose restriction
• Glucose restriction promotes the formation of oxidative muscle fibers
• Glucose restriction alters protein phosphorylation in skeletal muscle
• Perm1 may synergize AMPK and CaMK signaling under glucose restriction

## Graphical abstract

**Figure:**
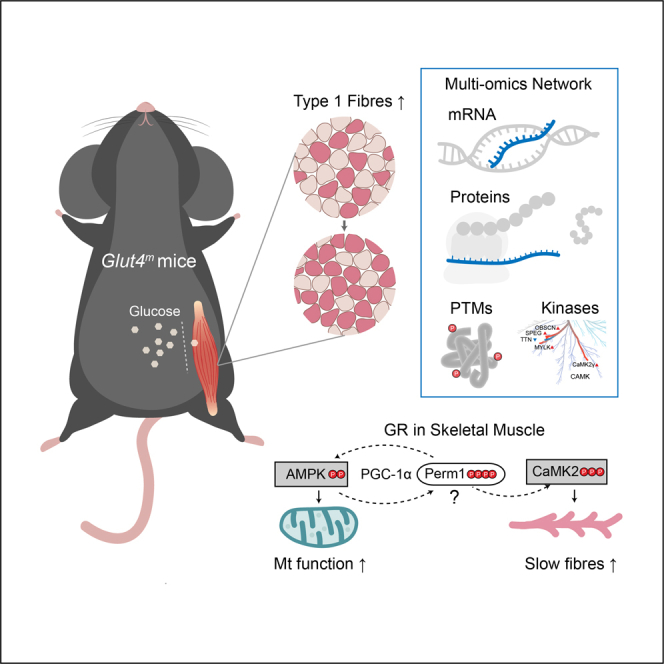

## Introduction

Skeletal muscle is a highly plastic organ responding to changing physical or metabolic states.^1^^,^^2^ As the main tissue of glucose storage and consumption, skeletal muscle plays a key role in the regulation of glucose homeostasis. There are two main classifications of muscle fibers. The type 1 fibers are enriched with mitochondria and mainly rely on oxidative metabolism and are adapted to continuous activities. The type 2 fibers (including 2A, 2B, and 2X) are characterized by glycolytic metabolism and are suitable for rapid explosive activities. In healthy individuals, skeletal muscle adapts to different physiological demands by altering energy metabolism patterns and muscle fiber types.^3^ In patients with obesity, insulin resistance, or diabetes, skeletal muscle metabolic flexibility is impaired, manifested primarily by increased glycolytic metabolism, fatty acid oxidation, and increased glycolytic fibers.^4^^,^^5^

Exercise and lifestyle interventions have been shown to provide significant benefits for individuals with metabolic diseases. For instance, calorie restriction improves mitochondrial metabolism and protects skeletal muscle from aging.^6^ Aerobic exercise increases oxidative phosphorylation in skeletal muscle^7^^,^^8^ and induces the secretion of beneficial myokines such as IL-6, Irisin, and Apelin that may help improve insulin sensitivity.^9^^,^^10^^,^^11^ Mechanically, calorie restriction and exercise promote AMPK activation and enhance mitochondrial function in skeletal muscles.^12^^,^^13^^,^^14^ Moreover, the cytoplasmic Ca^2+^ sensor calmodulin binds free Ca^2+^ and activates calmodulin-dependent protein kinase (CaMK), which in turn promotes the expression of genes associated with fast-to-slow fiber switch, mitochondrial production, and oxidative phosphorylation.^15^ Metabolic patterns play a fundamental role in differentiating muscle fiber types, making them a major factor in regulating muscle fiber type as well.^3^^,^^14^ However, there are complex molecular signaling mechanisms involved in the beneficial effects of exercise or calorie restriction, including transcriptional, translational, and post-translational modifications, which need further exploration. Mechanisms by which alterations in metabolic substrates affect muscle fiber composition remain unclear.

In this study, a mouse model of glucose restriction in skeletal muscle was constructed by mutating the glucose transporter 4 (GLUT4). GLUT4 is the only insulin-sensitive glucose transporter that is mainly expressed in skeletal muscle and adipose tissues.^16^ Based on the high structural conservation of the glucose transporter family,^17^^,^^18^^,^^19^ we introduced a glutamine-to-leucine mutant in the 5th transmembrane segment (TM5) of mice GLUT4 (GLUT4 p.Q177L), resulting in blocked substrate binding and the consequent glucose restriction in skeletal muscles. Compared to gene knockout, a GLUT4 mutation allows for the retention of protein expression and partial function, making it a more accurate representation of the “blocked” GLUT4 function observed in cases of insulin resistance. Increased slow muscle fibers and enhanced mitochondrial oxidative capacity were notable in the GLUT4 mutant (*Glut4*^*m*^) mice, which were beneficial to skeletal muscle metabolism. We performed a multi-omics analysis, including transcriptomic, proteomic, and phosphoproteomic on the skeletal muscle of *Glut4*^*m*^ mice at different growth stages and under different exercise states, deepening our knowledge of how GR benefits skeletal muscle and providing potential new targets for drug development.

## Results

### GLUT4 p.Q177L mutant limits insulin-stimulated glucose uptake in mice

**Figure 1:**
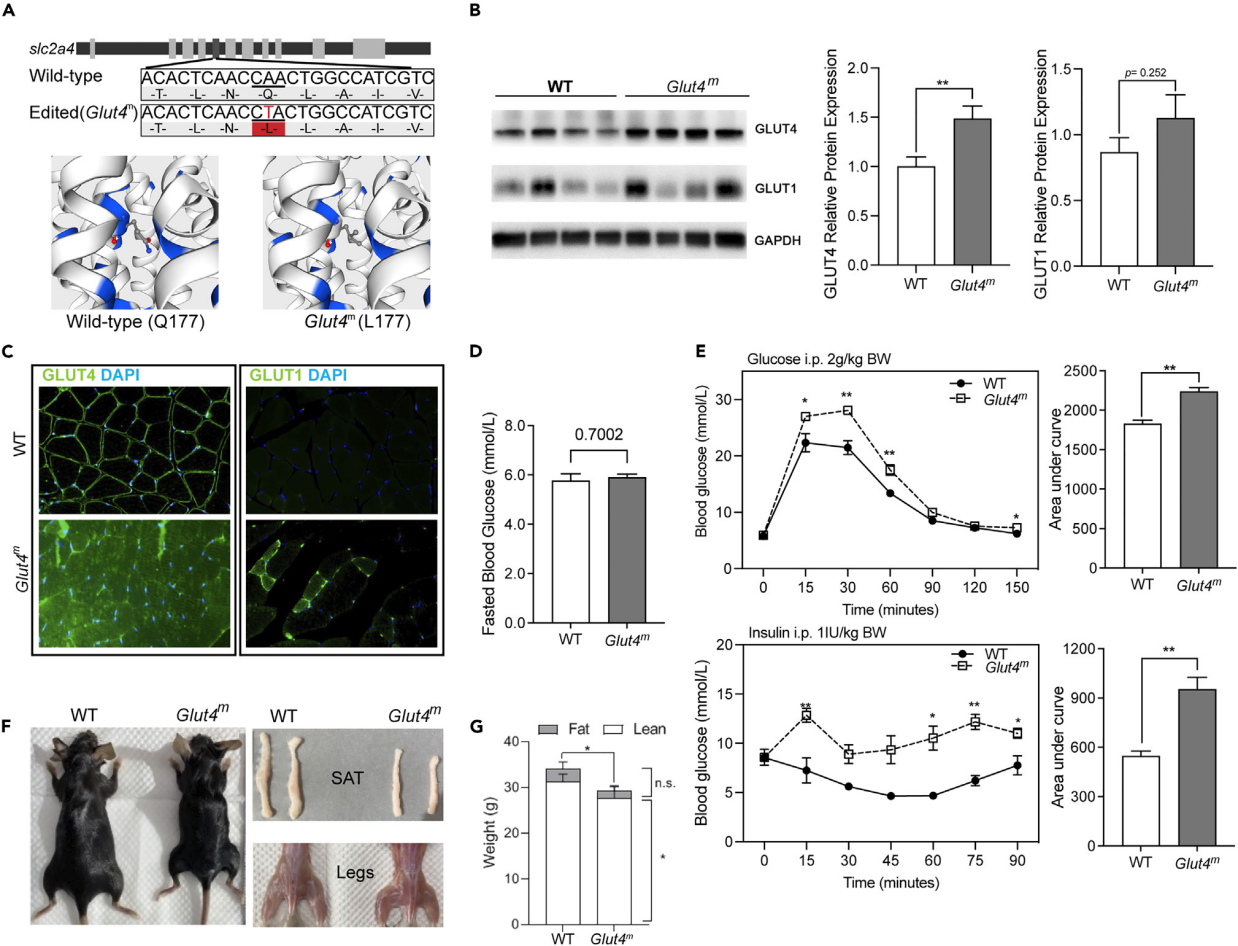
Structure, expression and effects on glucose disposal of the GLUT4 p.Q177L mutant (A) A→T transversion in exon 5 of the mouse *slc2a4* gene was manipulated by CRISPR/Cas9 system and the residue of glutamine was replaced with a lysine residue. (B) Immunoblotting analysis of GLUT4 and GLUT1 in muscle samples from WT and *Glut4*^*m*^ mice. (C) Immunofluorescence staining of GLUT4 and GLUT1 in mice gastrocnemius, showing the distinct distribution of the two glucose transporters in WT and *Glut4*^*m*^ mice. (D) Fasted blood glucose level of WT and *Glut4*^*m*^ mice. (E) Glucose tolerance test (glucose ip. 2 g/kg bw) and insulin tolerance test (insulin ip.1 IU/kg bw) in WT and *Glut4*^*m*^ mice. The statistical results of the area under the blood glucose curve of GTT and ITT experiments were displayed next to it. (F) Anatomy observation showed that *Glut4*^*m*^ mice have smaller body sizes and less fat deposition than those from WT mice. (G) Magnetic resonance analysis of body composition of WT and *Glut4*^*m*^ mice. *Glut4*^*m*^ mice had significantly lower body weight and fat mass than those from WT mice (p < 0.05). SAT: subcutaneous adipose tissue. Data are represented as mean ± SEM. ∗p *<* 0.05, ∗∗p < 0.01, n.s. not significant. See also Figures S1–S4.

GLUT4 p.Q177L mutant mice (*Glut4*^*m*^) were generated through CRISPR/Cas9-guided genetic editing that targeted the exon 5 of the *slc2a4* gene (coding GLUT4), which switches Gln177 to Leu177 in mice Glut4 protein (Figures 1A and S1). We detected the relative mRNA expression of multiple glucose transporters in their skeletal muscle through qPCR. The expression of *Glut1*, *Glut4*, and *Glut2* in *Glut4*^*m*^ were significantly higher than that of WT, among which *Glut1* and *Glut4* are the major glucose transporters in skeletal muscle (Figure S3). Immunoblotting analysis showed that protein expression of GLUT4 was significantly increased in *Glut4*^*m*^ skeletal muscle (Figures 1B and S2). Protein expression of the secondary glucose transporter, GLUT1, was slightly higher in *Glut4*^*m*^ skeletal muscle but did not reach statistical significance (Figures 1B and S2). To determine the cellular location of GLUT4 and GLUT1 in skeletal muscle, we assessed immunofluorescence (IF) staining of the two glucose transporters in paraffin-embedded gastrocnemius (Gas) sections (Figure 1C). Clearly, GLUT4 protein was predominantly located on the cell membrane, where it performs its function, in Gas sections from WT mice. Interestingly, the strong GLUT4 fluorescence intensity was found in the cytoplasm of Gas sections from *Glut4*^*m*^ mice, which is in line with the result that GLUT4 protein abundance was higher in *Glut4*^*m*^ muscle. GLUT1 is believed to control basal glucose uptake in various tissues including skeletal muscles. The fluorescence signal of GLUT1 was almost undetectable in WT Gas, which was in agreement with the fact that GLUT1 is not the predominant glucose transporter in skeletal muscle. In contrast, GLUT1 fluorescence was detected on the cell membrane in *Glut4*^*m*^ Gas sections and was selectively highly expressed in certain muscle fibers (Figure 1C), which may complement the function of mutant GLUT4 protein in some muscle fibers to ensure glucose supplement.

To further confirm that GLUT4 p.Q177L mutant affected glucose disposal, we performed a glucose tolerance test (GTT) and an insulin tolerance test (ITT) in *Glut4*^*m*^ and WT mice. Fasted blood glucose levels showed no significant difference between *Glut4*^*m*^ and WT (Figure 1D). After glucose supplement (2 g/kg, i.p.), blood glucose level of WT mice peaked (∼22.3 mmol/L) at 15 min and then gradually decreased (Figure 1E, upper-left panel). *Glut4*^*m*^ mice had significantly higher blood glucose level at 15 min (∼27.0 mmol/L) than that from WT mice and their blood glucose tended to keep increasing up to 30 min (Figure 1E, upper-left panel). Areas under *Glut4*^*m*^ GTT curves were also significantly higher than WT GTT curves (Figure 1E, upper-right panel). These data indicated that the GLUT4 p.Q177L mutation limits the rate of glucose distribution to GLUT4-expressing tissues, primarily skeletal muscle and adipose tissues, leading to a worse glucose handling capacity in mice. GLUT4 is the specific glucose transporter that responds to insulin, so we examined whether systemic insulin sensitivity was impaired by this mutation. To avoid hypoglycemia, mice were fasted for 8 h and received i.p. insulin 1 IU/kg body weight. *Glut4*^*m*^ and WT had almost the same initial blood glucose level, which was consistent with the result from GTT (Figure 1E, lower-left panel). 45 min after insulin injection, blood glucose of WT mice drop up to 50% of the basal level. However, the same dose of insulin led to an unexpectedly increased blood glucose level in *Glut4*^*m*^ in the first 15 min. Then the blood glucose level of *Glut4*^*m*^ recovered 30 min after injection and fluctuated slightly during the following time (Figure 1E, lower-left panel). The area under ITT curve of *Glut4*^*m*^ was almost 2-fold compared with WT (Figure 1E, lower-right panel), indicating that insulin-stimulated peripheral glucose disposal, which was mainly mediated by GLUT4, was significantly inhibited in *Glut4*^*m*^ mice.

Additionally, *Glut4*^*m*^ mice had smaller body sizes than age- and sex-matched WT mice, regardless of age (Figures 1F and S3). At different growth stages, *Glut4*^*m*^ mice had significantly larger heart weights as a percentage of their body weight. Liver weight percentages of *Glut4*^*m*^ mice were lower at 12 weeks but higher at 36 weeks compared to WT mice (Figure S3). We measured their body composition using nuclear magnetic resonance (NMR). NMR results were consistent with the anatomical observation that *Glut4*^*m*^ mice had significantly lower body weight and fat mass (Figure 1G). This result also matched the fact that GLUT4 dominates insulin-simulated glucose uptake in both skeletal muscle and adipose tissue. Together, we supposed that the GLUT4 mutant limited the glucose entry into skeletal muscle and adipose tissues, and restricted their development.

### GR promoted the formation of slow muscle fibers

**Figure 2:**
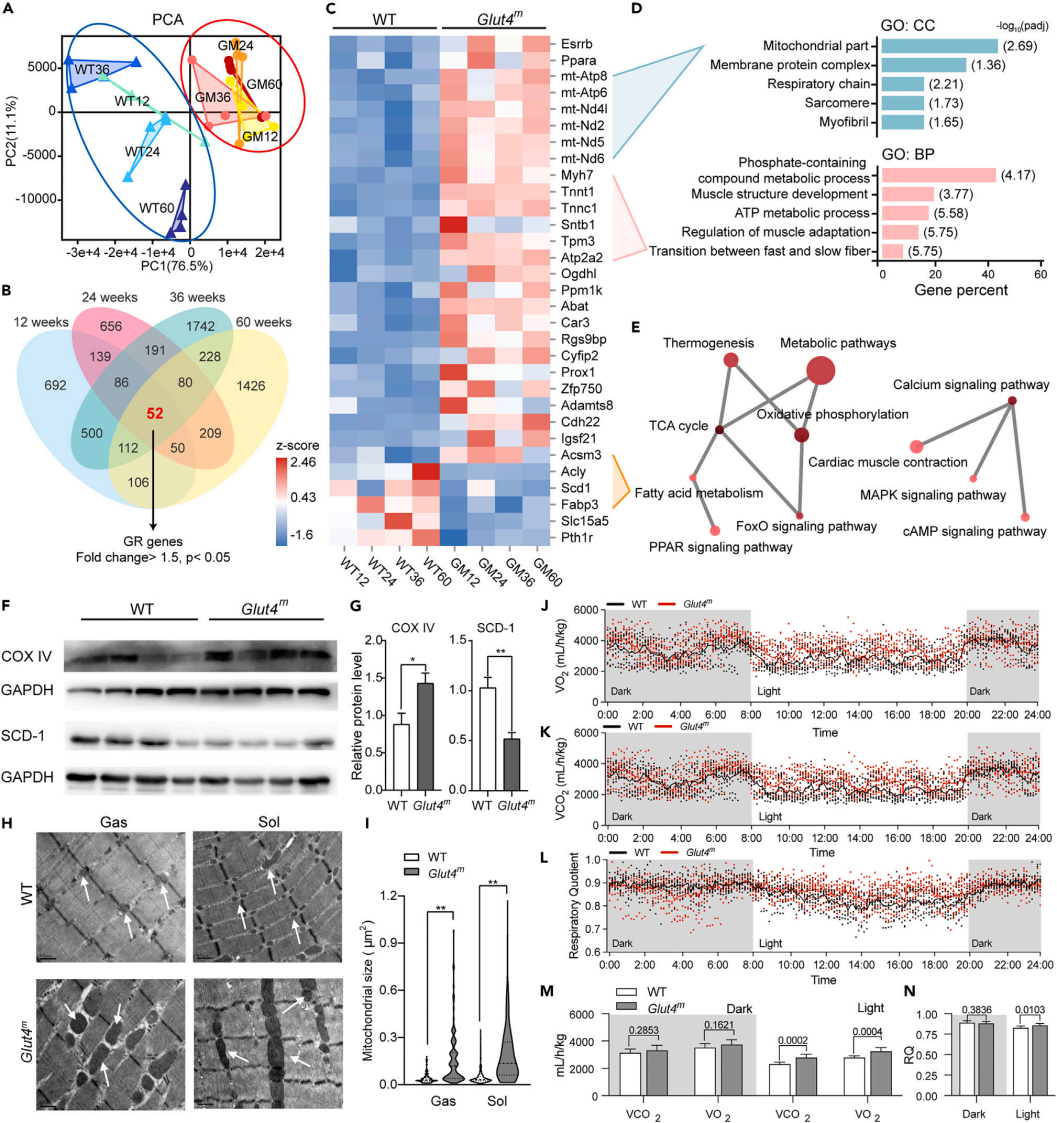
Transcriptome responses of glucose restriction and enhanced mitochondrial function in *Glut4*^*m*^ mice (A) Principal component analysis (PCA) indicated closed clustering of samples from *Glut4*^*m*^ mice at the different development stages and dispersed clustering of samples from WT mice at different ages (12, 24, 36, and 60 weeks). (B) Venn analysis of differentially expressed genes comparing Glut4^m^ mice and WT mice within each age (threshold: p < 0.05 and fold change> 1.5). 52 genes were differentially expressed in all ages of mice and were considered the major glucose-restriction-response genes (GR genes). (C) Heatmap of GR-responsive genes. (D) Gene ontology analysis of cellular components (GO: CC, blue) and biological processes (GO: BP, pink) of GR genes. (E) KEGG pathway enrichment network of GR genes. (F) Immunoblotting of COX IV and SCD-1. (G) Density statistic of immunoblotting images. (H) Representative electron micrographs of the gastrocnemius (Gas) and soleus (Sol), mitochondria were pointed out by white arrows. (I) Gas and Sol samples from *Glut4*^*m*^ mice had larger interfibrillar mitochondria with complete structure, indicating their mitochondrial function was enhanced under GR. (J–L) 24H *in vivo* indirect calorimetry analyses of WT (n = 8) and *Glut4*^*m*^ (n = 10) mice at 12 weeks of age. (M) Statistics of oxygen consumption (VO_2_) and carbon dioxide production (VCO_2_) in dark and light periods. (N) Statistic of respiratory quotient (RQ) value in dark and light periods. Data are represented as mean ± SEM. ∗p < 0.05, ∗∗p < 0.01, n.s. not significant. See also Tables S1 and S2 and Figure S7.

To gain further insights into the long-term influences on the skeletal muscle caused by GR, we performed transcriptome analysis in Gas muscle from both WT and *Glut4*^*m*^ mice at four growth stages with daily activity, including young (12 weeks), adult (24 weeks), old (36 weeks) and very old (60 weeks) stages. Principal component analysis (PCA) showed that *Glut4*^*m*^ samples on different development stages clustered together and were clearly separated from WT samples, indicating that skeletal transcriptome features were strongly influenced by GR (Figure 2A). The differentially expressed genes (DEGs) were screened by the criteria of p < 0.05 and fold change> 2 and 1737, 1463, 2991, and 2263 genes were identified as DEGs between both mice at the young, adult, old, and very-old ages, respectively.

Venn’s analysis of DEGs in four age groups showed that 52 genes were overlapped, in which 31 of exhibited a consistent trend in all comparisons (Figure 2B). These genes might be the major GR-responsive genes (GR genes) in skeletal muscle, regardless of age. Within these GR genes, 26 were upregulated in *Glut4*^*m*^ mice at all developmental stages, including slow fiber genes *Myh7*, *Atp2a2*, slow skeletal type troponin (*Tnnc1* and *Tnnt1*), mitochondrial genes, and important transcriptional regulators, *Esrrb* and *Ppara*. Five genes were observed to be downregulated in response to GR, including *Acly*, *Scd1*, and *Fabp3* involved in fatty acid metabolism (Figure 2C).

Gene ontology (GO) analysis showed that the GR genes were mainly mitochondrial-related genes or the component of muscle fibers (Figure 2D; Table S1). These genes were involved in muscle structure development, the transition between fast and slow fibers and ATP metabolic processes, suggesting that GR in *Glut4*^*m*^ muscle enhanced the mitochondrial function and muscle fiber transition. Almost half of the GR-responsive genes were related to the phosphate-containing compound metabolic process (Figure 2D). KEGG pathway analysis showed that GR genes are enriched in metabolic pathways, including oxidative phosphorylation, TCA cycle and fatty acid metabolism (Figure 2E; Table S1). In line with the transcriptome data, the protein level of SCD-1 in *Glut4*^*m*^ muscles was significantly lower than WT muscles, suggesting decreased fatty acid synthesis (Figures 2F and 2G).

Series tests of clusters were conducted using the STEM software^20^ to study the age-related gene expression patterns. RNA-seq data from two groups of mice at 4 ages were classified into 20 profiles (Figure S5). In WT samples, the expression of 181 genes continued to decline (profile 0) and 453 genes continued to increase (profile 19) with age (Figure S6A). In *Glut4*^*m*^ samples, 189 and 244 genes showed a trend of consistently decreasing (profile 0) or increasing (profile 19) expression (Figure S6B). Genes with the same expression pattern in both groups might be associated with skeletal muscle aging. In both groups, genes whose expression decreased with age were enriched in cancer-associated pathways (Figures S6C and S6D). In particular, the expression of WNT inhibitory factor 1 (*Wif1*), a canonical tumor suppressor, and insulin receptor substrate 2 (*Irs2*), were decreased with age in both *Glut4*^*m*^ and WT mice (Figure S6E). The age-dependent upregulated pathways in *Glut4*^*m*^ and WT mice were hematopoietic cell lineage (Figures S6C and S6D). Colony stimulating factor 3 receptor (*Csf3r*) which plays an important role in neutrophilic lineage proliferation and differentiation was one of the shared genes of profile 0 of *Glut4*^*m*^ and WT mice (Figure S6E). The expression patterns of 7 genes (*Abcc8, Mrap, Diras1, Zkscan16, 6720489N17Rik, Sv2c, and Trmt10a*) were completely opposite in the two groups of mice (Figure S6E) and these genes may be a future focus for research on aging and GR in skeletal muscle.

### GR enhanced the mitochondrial function in muscle of *Glut4*^*m*^ mice

The protein content of the mitochondrial respiratory chain enzyme, cytochrome *c* oxidase (COX IV) was significantly increased in *Glut4*^*m*^ muscles (Figures 2F and 2G). We further analyzed the mitochondrial morphology of gastrocnemius (Gas) and soleus (Sol) from *Glut4*^*m*^ and WT mice through transmission electron microscopy (TEM). Both Gas and Sol samples from WT mice had complete and orderly arranged sarcomeres and small interfibrillar (IMF) mitochondria near the Z-lines (Figure 2H top). On contrary, *Glut4*^*m*^ samples have notably enlarged and clustered IMF mitochondria which even interfered with myofibril alignment (Figure 2H bottom). Mitochondria size was calculated based on the TEM and data showed that the average mitochondrial size of *Glut4*^*m*^ mice was about 4-times larger than those from WT mice, in both Gas and Sol samples (Figure 2I). Large mitochondria in *Glut4*^*m*^ muscles had dense cristae and a complete outer membrane, illustrating that these enlarged mitochondria were healthy rather than injured or swelling (Figures 2H and S7).

To further investigate the effect of GR on the energy metabolism of skeletal muscle in both groups of mice, we used an indirect calorimetry system to continuously record their oxygen consumption and carbon dioxide production in 24 h. *Glut4*^*m*^ mice had higher oxygen consumption and carbon dioxide production than WT (Figures 2J and 2K), especially during the light period (8:00 a.m. to 20:00 p.m.) (Figure 2M). Consistent with the nocturnal lifestyle of mice, WT mice were more active during the dark period, while having decreased metabolism, oxygen consumption, and carbon dioxide production during the light period. However, *Glut4*^*m*^ mice seem to maintain a relatively high metabolic rate^21^ during the light period. Interestingly, the respiratory quotient (RQ) of *Glut4*^*m*^ mice was slightly lower than WT in the dark period, while higher in the light period (Figures 2L and 2N). This result probably reflected the metabolic adaptations to cope with reduced glucose availability and maintain energy homeostasis through the utilization of alternative substrates. *Glut4*^*m*^ mice might be utilizing more fatty acids in the dark period, and utilizing more amino acids or lactate in the light period.

### GR increased slow fibers in gastrocnemius and soleus of *Glut4*^*m*^ mice

**Figure 3:**
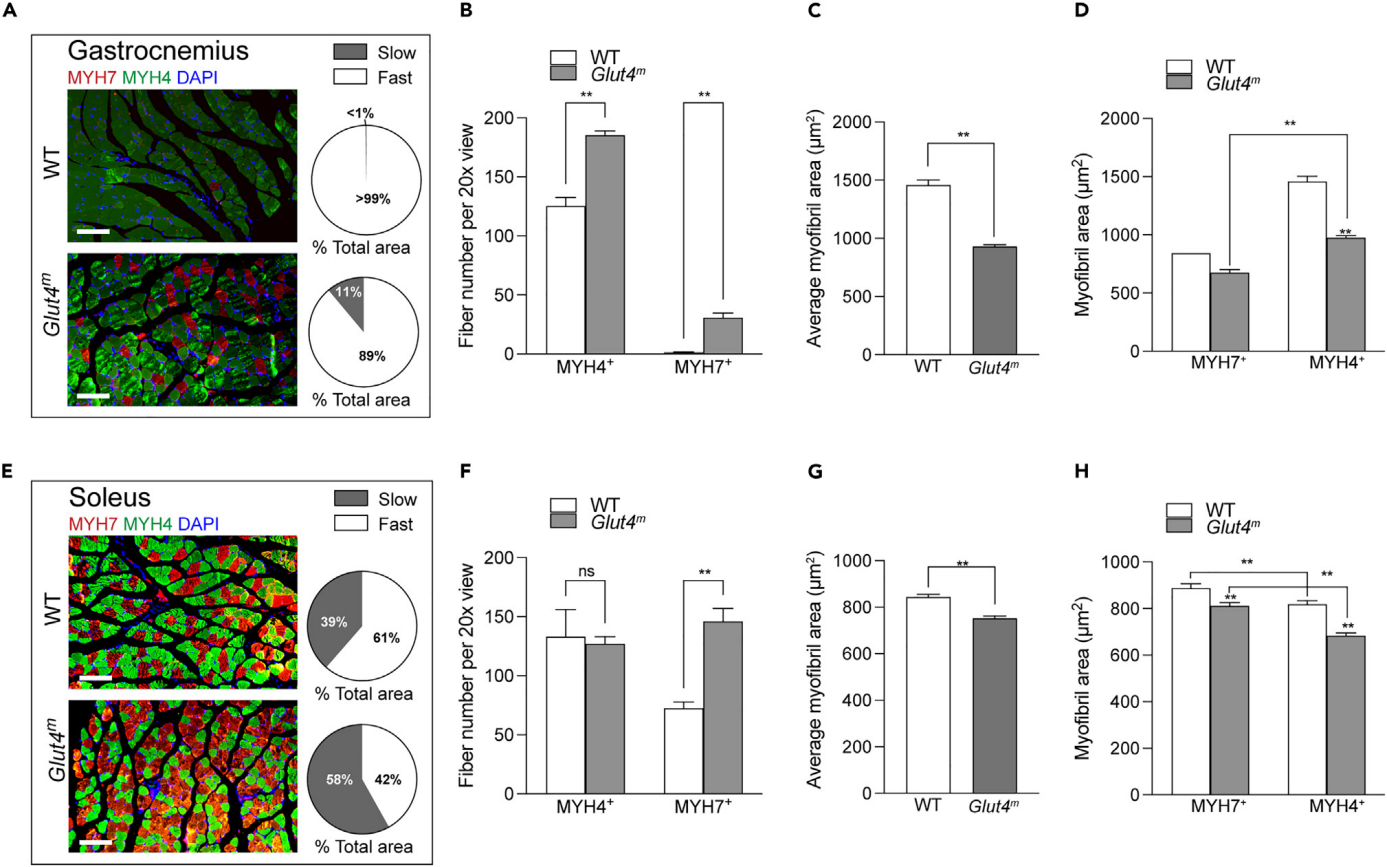
Increased slow fibers in muscles of *Glut4*^*m*^ mice Immunofluorescence staining (IF) of MYH7 and MYH4 and statistics of slow and fast fibers in gastrocnemius samples (A–D) and soleus samples (E and F). (A and E) Representative IF images and statistics of the proportion of MHY4^+^ (fast fiber) and MYH7^+^ (slow fiber) areas. N = 4 per group, scale bars are 100 μm. (B and F) The number of each type of fibers in each 20x view (n = 4 per group). (C and G) Average myofibril area, no distinction between fast and slow fibers. (D and H) Average myofibril area of MYH4^+^ and MYH7^+^ fibers, respectively. Statistics of single muscle fiber sizes in IF images: 526 and 390 fibers were calculated in Sol samples from *Glut4*^*m*^ and WT mice; 660 and 244 fibers were calculated in Gas samples from *Glut4*^*m*^ and WT mice. Data are represented as mean ± SEM. ∗∗p < 0.01, ns, not significant.

Skeletal muscle is a highly flexible organ in which fiber composition shifts with the metabolic state. GR leads to significantly enhanced mitochondrial function, so we speculated a corresponding change in muscle fiber type in *Glut4*^*m*^ mice. We performed IF staining for MYH7 and MYH4, which are hallmarks for oxidative slow-twitch type 1 fibers and glycolytic fast-twitch type 2b fibers respectively, in Gas and Sol from both mice (Figures 3A and 3B). As expected, *Glut4*^*m*^ mice had clearly increased the number of MYH7^+^ slow fibers in both Gas and Sol samples, when compared to those from WT (Figure 3C).

We also measured the individual muscle fiber size in IF staining images. The average muscle fiber size in Gas from WT mice was about 1.5 times larger than Gas from *Glut4*^*m*^ mice (p < 0.01). The difference in Sol was slightly smaller between the two groups (p < 0.01), but still significant (Figure 3C). When calculating the size of fast and slow fibers, we found that although both fibers in *Glut4*^*m*^ muscles had shrunken individual sizes (Figure 3C), the proportion of the total area of slow fibers was still significantly higher in *Glut4*^*m*^ muscles than in WT muscles (Figures 3A and 3B). Statistic results illustrated that there was a fast-to-slow fiber-type change in skeletal muscles under GR.

### GR promotes slow fiber- and myokine-related gene expression throughout the lifespan

**Figure 4:**
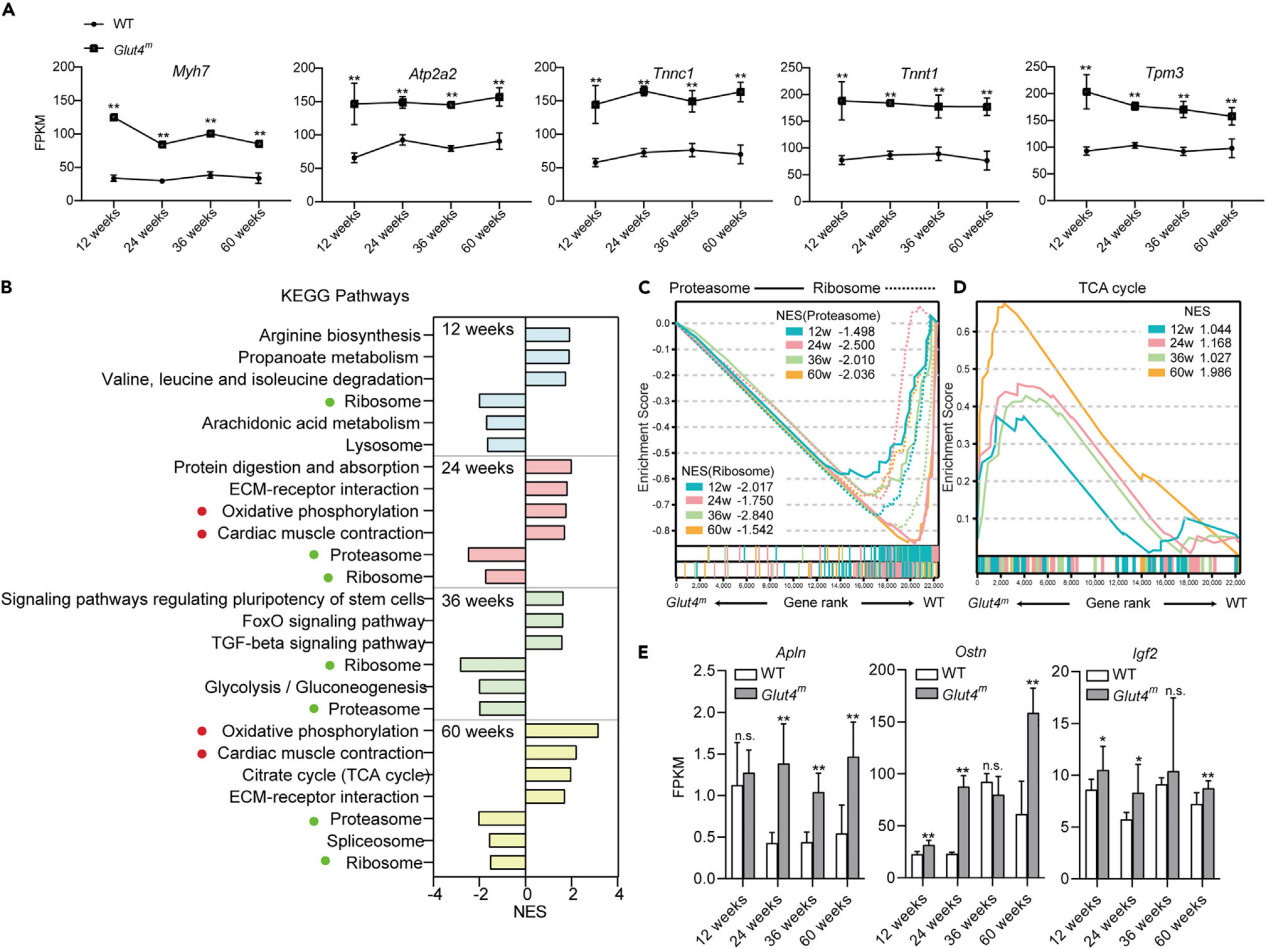
Transcriptome differences between *Glut4*^*m*^ and WT mice at different ages (A) mRNA expression of the core genes involved in slow fiber formation. (B) Top GSEA enriched KEGG pathways comparing *Glut4*^*m*^ and WT mice of different ages. A positive normalized enrichment score (NES) indicated that the pathway was up-regulated in *Glut4*^*m*^ samples, while a negative NES indicated down-regulated pathways. Key pathways that positively or negatively responded to GR in all age groups were noted with red or green plots. (C and D) GSEA enrichment map. The result of different ages was represented by different colors and NES scores were shown respectively. (E) mRNA expression *Apln, Ostn* and *Igf2* at different ages. Data are represented as mean ± SEM. ∗FDR<0.05, ∗∗FDR<0.01, n.s. not significant. See also Table S3, Figures S5 and S6.

Next, we analyzed the difference in biological pathways between *Glut4*^*m*^ mice and WT mice at four ages by gene set enrichment analysis (GSEA) using RNA-seq data. We observed the significantly upregulated cardiac muscle-related pathways during all developmental stages in *Glut4*^*m*^ mice. Core genes in these pathways included *Myh7*, *Atp2a2*, *Tnnc1*, *Tnnt1*, and *Tpm3* that function in both cardiac muscles and slow skeletal muscles (Figure 4A), consisting of the fact that *Glut4*^*m*^ muscle contained more slow fibers, regardless of age. Additionally, these genes did not show significant expression trends with age, indicating their relatively stable expression in both groups (Figure S5) Ribosome pathway and proteasome pathway were significantly downregulated in *Glut4*^*m*^ muscles at four different ages (Figures 4B and 4C), suggesting that protein synthesis and degradation cycling might be limited by GR. GR enhanced the TCA cycle in muscle in an age-dependent manner that normalized enrichment scores (NES) of the TCA cycle increased with age at 12, 24, and 60 weeks (Figure 4D). There were interesting findings when we focused on the older age groups. At 36 weeks, *Glut4*^*m*^ mice had stronger TGF-beta signaling, FoxO signaling, and enhanced signaling pathways regulating pluripotency of stem cells, and at 60 weeks, they still had more active oxidative phosphorylation and TCA cycling than WT mice, indicating that *Glut4*^*m*^ mice at old ages may retain more proliferative capacity and metabolic activity than WT mice (Figure 4B).

Skeletal muscle secretes a panel of myokines that regulates muscle function. To identify GR-induced myokines, we predicted genes encoding secreted proteins in transcriptome results using The Human Protein Atlas database (https://www.proteinatlas.org/). 2425 genes were annotated to encode secreted proteins and 781 of them showed a significant difference between *Glut4*^*m*^ and WT mice in at least one age stage (Table S3). Interestingly, some exercise-induced myokines such as apelin (encoded by *Apln*) and musclin (encoded by *Ostn*)^11^^,^^22^ were upregulated at mRNA levels in *Glut4*^*m*^ mice at different ages (Figure 4E). GR also induced a significantly increased expression of insulin-like growth factor 2 (*Igf2*) (Figure 4E) and angiogenic factors including *Vegfa, Vegfb* and *Angpt1* (Table S3), indicating that these secreted factors might be involved in the regulation of muscle development in the context of GR.

### Low-intensity treadmill training increased slow fibers in *Glut4*^*m*^ mice

**Figure 5:**
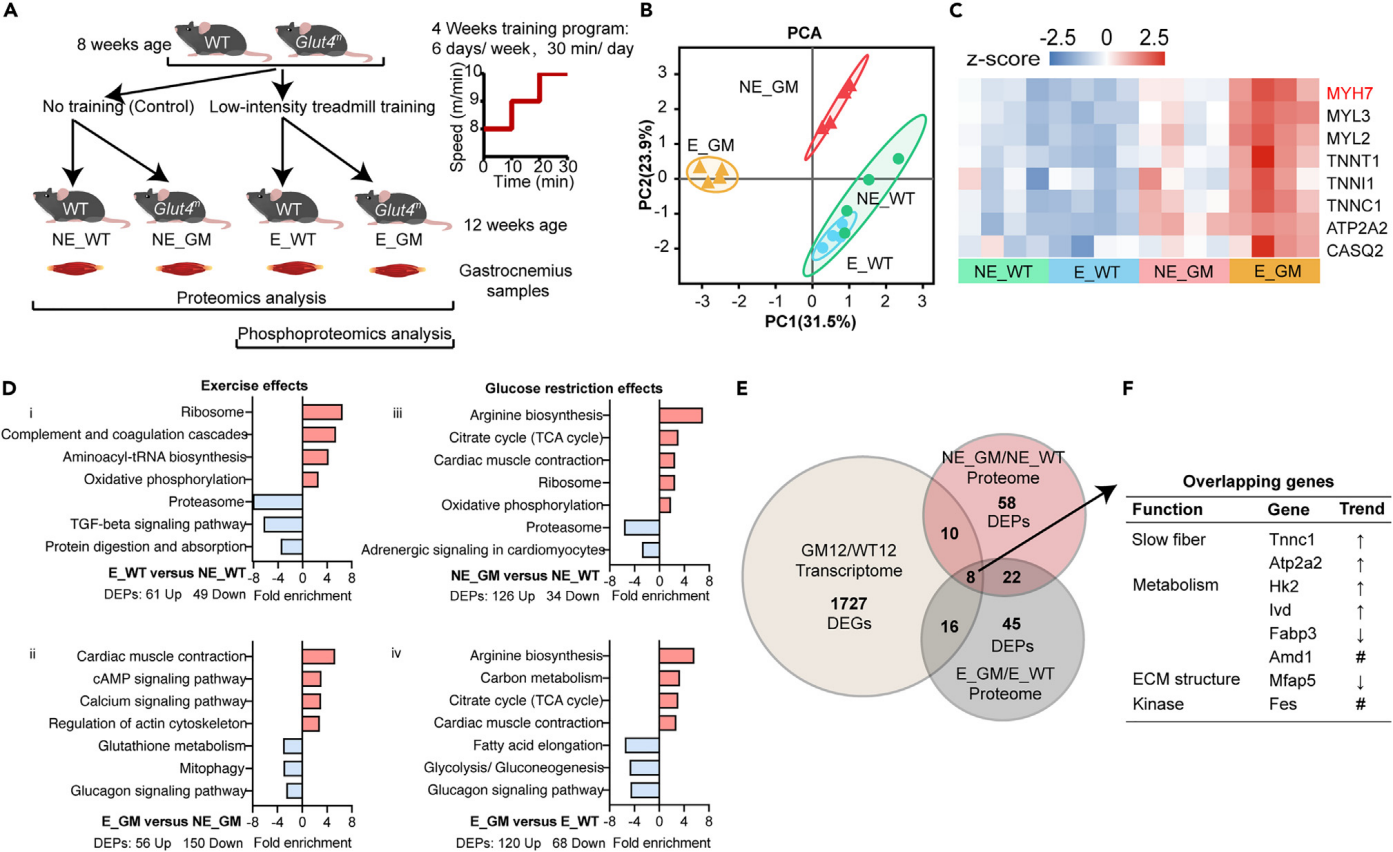
Effects of GR and low-intensity treadmill training on skeletal muscle proteome (A) Study design and the training program of low-intensity treadmill training (LIT). (B) Principal component analysis (PCA) of the proteome data of four groups of mice, including untrained WT mice (NE_WT), untrained *Glut4*^*m*^ mice (NE_GM), LIT-trained WT mice (E_WT) and LIT-trained *Glut4*^*m*^ mice (E_GM), n = 4 per group. (C) Heatmap showing the expression of slow fiber-associated proteins in four groups. Data were normalized using a *Z* score, with blue color indicating low expression and red color indicating high expression. (D) Significantly enriched KEGG pathways of differentially expressed proteins (p *<* 0.05, fold change> 1.2) comparing four experiment groups. Red bars indicated up-regulated pathways; blue bars indicated down-regulated pathways. (E) Venn map of differentially expressed molecules (*Glut4*^*m*^ versus WT) identified at transcriptome level (p *<* 0.05, fold change> 1.5) and proteome level (p *<* 0.05, fold change> 1.2). (F) Function annotation of 8 overlapping molecules from E. ↑, increased mRNA expression and protein level in *Glut4*^*m*^ mice; ↓, decreased mRNA expression and protein level in *Glut4*^*m*^ mice; # inconsistent difference at mRNA and protein level between *Glut4*^*m*^ and WT. DEGs, differentially expressed genes; DEPs differentially expressed proteins. See also Tables S4 and S5.

Appropriate exercise has positive effects on skeletal muscle health and low-intensity training is sufficient to affect muscle physiology.^23^ We next tried to figure out whether *Glut4*^*m*^ mice respond the same as WT mice to low-intensity training, and whether the beneficial changes in *Glut4*^*m*^ muscles have a similar mechanism to exercise effects. In order to simulate mild exercise, like walking, we choose treadmill training to flexibly control exercise intensity. According to previous research,^23^ low-intensity treadmill training (LIT) in this study was set at 8–10 m/min with 0° slop, 20–30 min per day, 6 days per week (Figure 5A). The 4-week LIT consisted of 1-week adaptive training (8 m/min, 20 min per day) and 3-week regular training (8–10 m/min gradually, 30 min per day). After the last training, Gas samples were collected and stored in liquid nitrogen immediately to preserve post-translational information.

We performed TMT-labelled mass spectrometry analysis of Gas samples from LIT-trained mice (E_WT and E_GM, n = 4 per group), and matched untrained mice (NE_WT and NE_GM, n = 4 per group). 2571 proteins were identified and 2191 proteins were further quantified. PCA analysis revealed the overall proteomic difference between the 4 groups, in which the first two principal components (PC1 and PC2) accounted for 55.4% of the total variation (Figure 5B). There was an overlapped distribution on the PCA map between WT samples with and without LIT. On the contrary, there was a wide separation between *Glut4*^*m*^ samples with and without LIT. The widest separation was found between the E_GM group and WT groups, with the NE_GM group located between them on the PCA map. PCA results indicated that LIT in this experiment significantly affects *Glut4*^*m*^ muscles.

Protein levels of a panel of slow fiber-associated molecules (MYH7, MYL3, MYL2, TNNT1, TNNI1, TNNC1, ATP2A2, and CASQ2) were highest in E_GM, followed by that in NE_GM, while the abundance of these proteins showed no difference between E_WT and NE_WT (Figure 5C). This shows that the intensity of LIT is different for the two groups of mice. In *Glut4*^*m*^ mice, this low-intensity stimulation significantly increased slow fibers in Gas. The highest level of slow muscle-related proteins in the E_GM group also indicated that GR and LIT might have a synergistic effect on promoting slow muscle formation.

Consistent with our expectations, LIT induced significant beneficial changes in skeletal muscle metabolism in WT mice. While not changing the composition of muscle fibers, LIT significantly enhanced oxidative phosphorylation in WT mice. Meanwhile, LIT significantly promoted protein translation and suppressed protein digestion in WT, as proved by the upregulated ribosome and aminoacyl-tRNA biosynthesis pathways, and downregulated proteasome and protein digestion and absorption pathways (Figure 5D i; Table S4). However, LIT brought entirely different effects in *Glut4*^*m*^ mice. LIT stimulated calcium signaling pathways and cAMP signaling pathways, which are key pathways regulating slow fiber formation. Correspondingly, the cardiac muscle contraction pathway and the regulation of actin cytoskeleton pathway were upregulated in the E_GM group than that in the NE_GM group, and the main contributing genes were related to slow skeletal muscle fibers. Glucagon signaling, glutathione metabolism and mitophagy pathways were downregulated in E_GM versus NE_GM (Figure 5D ii; Table S4).

### GR amplify the effect of LIT by enhancing TCA and arginine biosynthesis

When comparing the proteome of *Glut4*^*m*^ and WT mice without LIT, the enhanced oxidative metabolism and increased slow-twitch fibers were confirmed. GR upregulated arginine biosynthesis, TCA cycle, cardiac muscle contraction, ribosome and oxidative phosphorylation pathways, leading to slow fiber formation. Meanwhile, the adrenergic signaling pathway, which has been proven to trigger fast muscle formation, was downregulated in the NE_GM group compared with the NE_WT group. Proteasome activity also declined in NE_GM versus NE_WT, indicating that protein degradation in *Glut4*^*m*^ muscle was reduced (Figure 5D iii; Table S4).

When comparing *Glut4*^*m*^ mice and WT mice which were both trained by LIT, their difference in muscle fiber type and energy metabolism between E_GM and E_WT were broadened. There was significantly enhanced arginine biosynthesis, carbon metabolism and TCA cycle in LIT-trained *Glut4*^*m*^ muscles, whereas fatty acid elongation, gluconeogenesis and the glucagon signaling pathway were downregulated (Figure 5D iv; Table S4).

Moreover, there were 8 overlapping molecules that responded to GR in transcriptome data and proteome data, regardless of LIT (Figure 5E). The mRNA expression and protein level of slow fiber structure-associated molecules *Tnnc1* and *Atp2a2* were significantly higher in *Glut4*^*m*^ samples (Figure 5F). Hexokinase 2 (*Hk2*) that catalysis the first and rate-limiting step in glucose metabolism and Isovaleryl-CoA dehydrogenase (*Ivd*) involved in amino acid degradation, were also upregulated in *Glut4*^*m*^ mice. Fatty acid binding protein 3 (*Fabp3*) and microfibril associated protein 5 (*Mfap5*) were downregulated at mRNA and protein levels comparing *Glut4*^*m*^ with WT (Figure 5F). In aggregate, proteomic data demonstrate that GR has a profound effect on skeletal muscle metabolism and facilitates fiber type shift during LIT.

### GR alters protein phosphorylation in muscles

**Figure 6:**
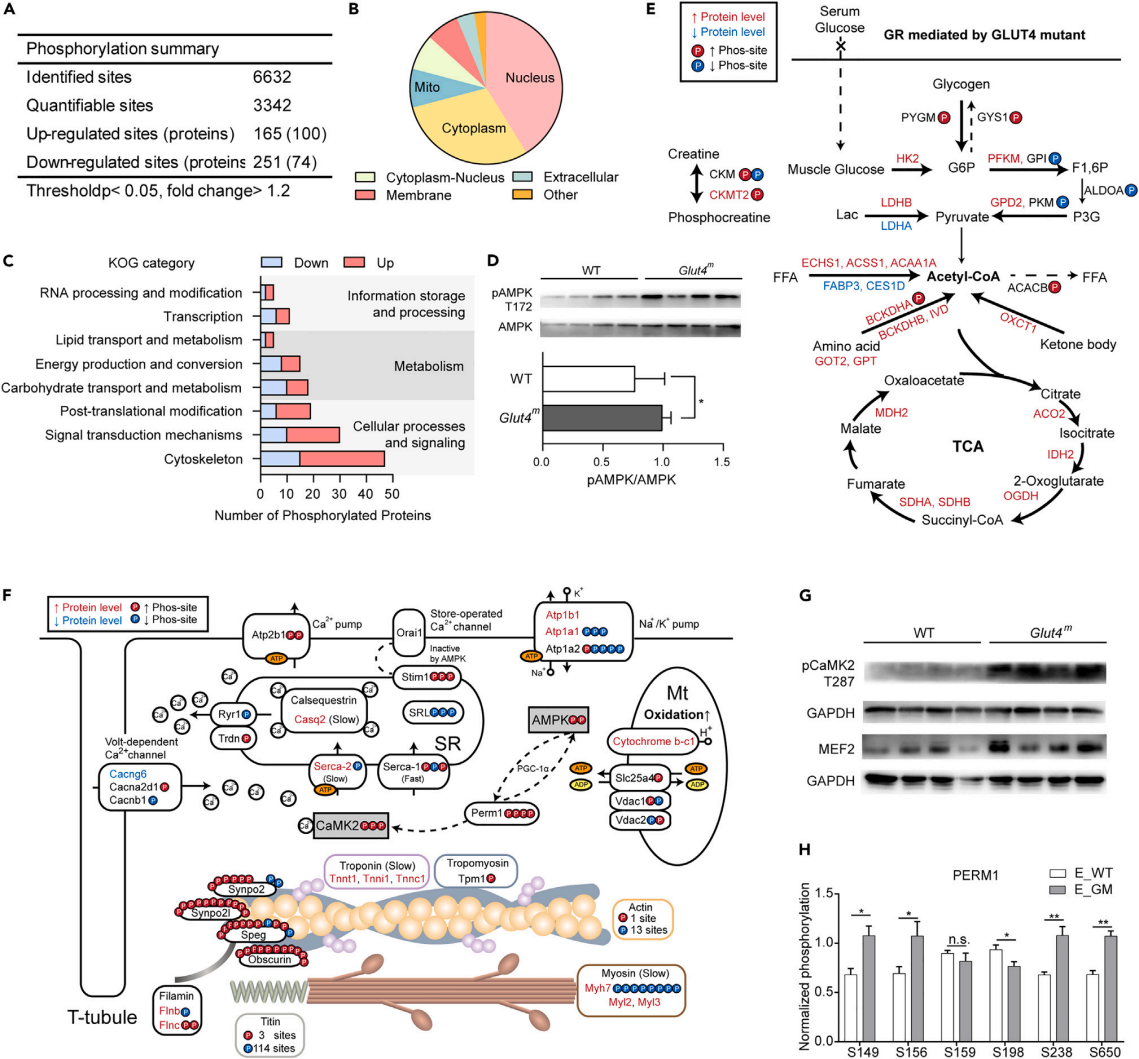
Phosphorylation changes in GR response (A) MS/MS identified and quantified phosphorylation sites. (B) Subcellular location of proteins with up- or down-regulated phosphorylation sites between *Glut4*^*m*^ and WT muscles. (C) Function category enrichment of proteins with different phosphorylation modifications indicated that the dominant difference between *Glut4*^*m*^ mice and WT mice at phosphorylation level was involved in cellular signaling, cytoskeleton structure, metabolic-related enzymes, and nucleus proteins that function in RNA processing and transcription. (D) Immunoblotting results of total AMPK and pAMPK (T172). The degree of activation of AMPK was shown as pAMPK/AMPK. (E) Metabolic enzymes with altered protein level or changed phosphorylation state. The bold arrow indicated activated metabolic processes while doted arrow indicated inhibited metabolic processes in *Glut4*^*m*^ muscles. (F) Differentially phosphorylated sites in myofibrillar proteins. The color of text and phosphorylation sites highlighted their significant up-regulation (red) or down-regulation (green). The number of differently phosphorylated sites in each protein was shown directly. (G) Immunoblotting results of pCaMK2 (T287) and MEF2, statistic results could be found in Figure S2. (H) Phosphoproteome identified 6 phosphorylation sites in PERM1, 5 of them were differently modified between E_GM and E_WT muscles. Data are represented as mean ± SEM. ∗p < 0.05, ∗∗p < 0.01, n.s. not significant. See also Tables S5 and S6.

Phosphorylation is an important post-translational modification that regulates cellular signal transduction and metabolic enzyme activity. In the transcriptome study, we noticed that the GR-responsive genes were enriched in phosphate-containing compound metabolic processes (Figure 2D). Further, in the proteomic study, we found that GR significantly increased muscle sensitivity to light. To characterize the global metabolic state and cell signaling in muscles with GR, we performed quantitative phosphoproteomic analysis on samples from LIT-trained *Glut4*^*m*^ and WT mice (E_GM and E_WT). We identified 6632 phosphorylation sites, of which 3342 sites were further quantified and normalized with protein abundance (Table S5). At the threshold p *<* 0.05 and fold change> 1.2, 165 phosphorylation sites of 100 proteins were upregulated and 251 sites on 74 proteins were downregulated in E_GM versus E_WT muscle (Figure 6A).

Proteins with differential phosphorylation modification were predicted to mainly locate in the nucleus, cytoplasm and mitochondria (Figure 6B). Functional classification based on the KOG (Clusters of orthologous groups for eukaryotic complete genomes) database suggested that these proteins were most enriched in the KOG category of information storage and processing, cellular processes and signaling, and metabolism (Figure 6C). Correspondingly, the top enriched KEGG pathways included metabolism pathways, cardiac muscle contraction and related pathways, and the calcium signaling pathway (Table S6).

### GR altered protein phosphorylation in AMPK signaling pathway and TCA cycle

As a consequence of GR, the energy sensor protein kinase AMPK was significantly more active in *Glut4*^*m*^ muscles (Figure 6D). Activation of AMPK can directly phosphorylate metabolic enzymes to regulate their activity, or regulate their expression through downstream transcription factors, thereby activating ATP production. Energy substrates, including glucose, glycogen, lactate, lipids, amino acids and ketone bodies in muscle from *Glut4*^*m*^ mice, were more efficiently utilized to meet cellular energy demand, and the changes in the related enzymes were shown in Figure 6E.

Glycogen synthesis and breakdown is a metabolic process that typically is regulated by phosphorylation modification on key enzymes. Glycogen synthase (GYS1) is inhibited when phosphorylated by AMPK while glycogen phosphorylase (PYGM), which catalyses glycogen breakdown to glucose, is activated when phosphorylated by AMPK. In E_GM muscles, both GYS1 and PYGM had elevated phosphorylation states, indicating that glycogen utilization was enhanced and its synthesis was weakened by GR.

Enzymes that produce pyruvate from carbohydrates showed increased expression or greater activity in E_GM muscles. HK2 catalyses the initial and rate-limiting step of glycolysis, and muscle-type phosphofructokinase (PFKM) that phosphorylate fructose 6-phosphate to fructose 1, 6-bisphosphate, was upregulated at the protein level in E_GM samples. Glucose-6-phosphate (GPI) was less phosphorylated in the E_GM group, meaning its more active than that in E_WT samples. Pyruvate kinase (PKM), a key glycolytic enzyme regulated by phosphorylation modification, had lower phosphorylation in E_GM samples. This sequence of changes suggested an enhanced pyruvate production in the glycolysis process. Lactate dehydrogenase (LDH) catalyses the interconversion of pyruvate and lactate in the post-glycolysis process and the LDH isomers containing more H subunit prefers to dehydrogenate lactate to pyruvate. In E_GM muscles, the protein level of its H subunit (LDHB) was significantly increased and its M subunit (LDHA) was significantly decreased, suggesting that the direction of this reaction tended to produce pyruvate from lactate. This suggests that *Glut4*^*m*^ mice utilized lactate more efficiently.

On the other hand, enzymes that catalyze lipid degradation (ECHS1, ACSS1 and ACAA1A), amino acid degradation (GOT2, GPT, BCKDHA, BCKDHB, IVD), and the rate-limiting enzyme catalyses ketone bodies to acetyl-CoA, the 3-oxoacid CoA-transferase 1 (OXCT1), were all upregulated at the protein level in E_GM muscles. In contrast, acetyl-CoA carboxylase (ACACB) which utilizes acetyl-CoA as the substrate for *de novo* lipogenesis, was inhibited by increased phosphorylation in E_GM muscles. These results suggested a more active acetyl-CoA-producing metabolism in muscles from *Glut4*^*m*^ mice.

Enzymes in the TCA cycle, including aconitase 2(ACO2), isocitrate dehydrogenase 2 (IDH2), oxoglutarate dehydrogenase (OGDH), succinate dehydrogenase (SDHB) and malate dehydrogenase 2 (MDH2) were all upregulated at protein level, pointing to enhanced TCA cycling in E_GM muscle Mitochondrial creatine kinase (CKMT2) that transfers high energy phosphate from mitochondria to creatine, was increased at protein level and more phosphorylated in E_GM (Figure 6E). Mitochondria proteins that mediate ATP production and export from mitochondria were differentially phosphorylated in response to GR, which would benefit cellular ATP supplement (Figure 6F).

From these results, we speculate that skeletal function adapts to GR by enhancing acetyl-CoA production and TCA cycle as well as attenuating anabolism.

### GR changed phosphorylation of myofibrillar proteins and calcium signaling pathway

KOG functional classification showed that over 40% of differential phosphorylation modification occurred on myofibrillar proteins (Figure 6C). In actin filaments, GR increased the phosphorylation of S186 in tropomyosin (TPM1) and T188 in actin (ACTA1), while it decreased the phosphorylation of 13 other modified sites in ACTA1 and S58 in slow-type troponin subunit Tnni1(Figure 6F). In myosin filaments, the protein levels of slow muscle-specific MYH7, MYL2 and MYL3 (Figures 5C and 6F) were increased by GR effect, but phosphorylation of MYH7 was significantly decreased at 8 modified sites by GR. As the key component in the assembly of sarcomere, titin (TTN) phosphorylation was dramatically altered responding to GR. TTN from E_GM muscles had 3 sites with increased phosphorylation and 114 modified sites with decreased phosphorylation (Figure 6F). GR significantly altered phosphorylation of proteins in the Z-disk. Striated muscle-enriched protein kinase (SPEG) and obscurin (OBSCN), which have kinase activity, were basically more phosphorylated under GR influence. Snaptopodin 2 (SYNPO2) and snaptopodin 2-like protein (SYNPO2L) that help assembly and stabilization of Z-lines were also more phosphorylated at most modified residues in E_GM muscles. Filamin subunit FLNB showed a decreased phosphorylation at S2478 while subunit FLNC showed increased phosphorylation at S1528 and S2123 (Figure 6F).

Proteins involved in calcium cycling and storage had differential protein abundance or phosphorylation modification. For instance, slow muscle-specific calcium store calsequestrin 2 (CASQ2) and slow muscle-specific sarcoplasmic/endoplasmic reticulum calcium ATPase 2 (SERCA2, also refers as ATP2A2) were higher expressed at the protein level in E_GM muscles. Apart from that, other sarcoplasmic/membrane calcium channels and calcium pumps had markable different phosphorylation sites between E_WT and E_GM muscles (Figure 6G), suggesting that the calcium signaling pathway might be also affected. Indeed, S311, S381 and S384 on CaMK2γ (CaMK2 subunit gamma) had significantly increased phosphorylation in E_GM muscles (Table S5). T287 phosphorylation in CaMK2γ was 1.3-folded in E_GM versus E_WT (p = 0.097, Table S5). To verify CaMK2 activation in *Glut4*^*m*^ muscles, we detected T287-specific phosphorylation of CaMK2 and protein expression of downstream transcription factor MEF2 that controls slow fiber formation. The immunoblotting results confirmed phospho-proteome data that *Glut4*^*m*^ samples had a significantly higher level of pCaMK2 and MEF2 than WT samples (Figure 6G), indicating that in the myogenesis progress of slow fibers in *Glut4*^*m*^ mice, the CaMK2-MEF2 signaling pathway was closely involved. In addition, the PPARGC1 and ESRR induced regulator in muscle 1 (PERM1) which was reported to regulate the expression of PPARGC1 and ESRR target genes and CaMK2 activation in muscle response to endurance training, showed significantly altered phosphorylation by GR effect (Figure 6H).

### Enrichment of CAMK group kinases in *Glut4*^*m*^ samples

**Figure 7:**
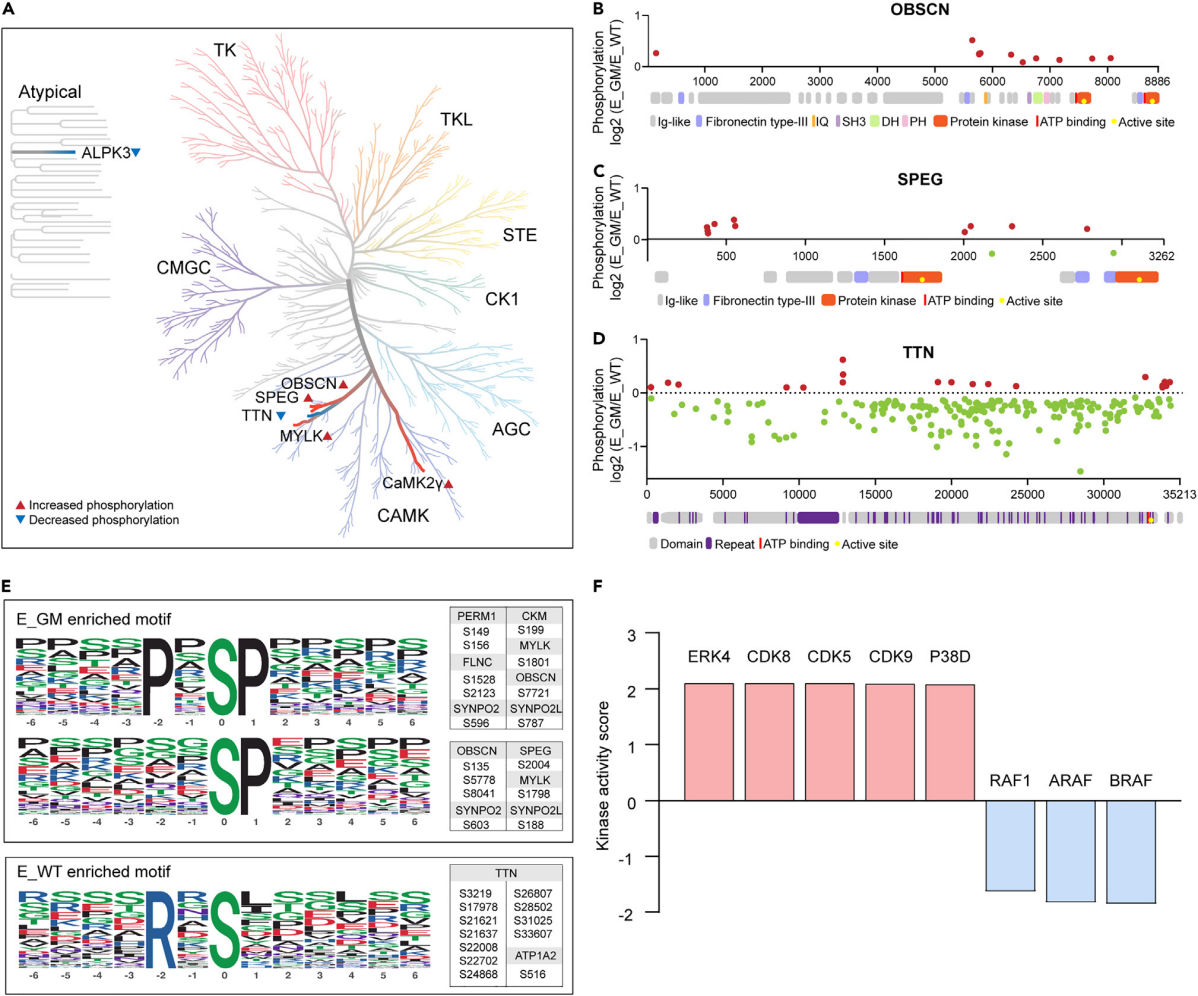
Motif enrichment and kinase activity analysis (A) Kinase with GR-induced alterations (p *<* 0.05) were plotted on a phylogenetic tree of human kinome (visualized by Coral http://phanstiel-lab.med.unc.edu/CORAL/). (B–D) Schematic representation of the protein domain of OBSCN (B), SPEG (C) and TTN (D) and phosphorylation status comparing E_GM with E_WT muscles. Modified sites with GR-induced increase (p *<* 0.05 and log2FC > 0) or decrease (p *<* 0.05 and log2FC < 0) were indicated with red or green dots. (E) Enriched phosphorylation motifs in E_GM and E_WT muscles and the major contributing sites with significant GR-induced difference. (F) Kinase prediction based on kinase activity enrichment analysis (KSEA). Kinase activity score >0 suggests that the kinase tends to be more active in E_GM muscles and kinase activity score <0 suggests that the kinase tends to be more active in E_WT muscles. See also Table S5.

Next, we wanted to understand which kinase might be responsible for those GR-induced phosphorylation changes. According to The Mouse Kinome (S Caenepeel et al.),^24^ there are currently 651 known mouse kinases. The phosphorylation status of a kinase can reflect its activity, so we first scanned kinases with altered phosphorylation sites in our phosphoproteome data. Over the 3342 phosphorylation sites we quantified, 942 sites were located on 33 kinases, of which 13 kinases belong to the CAMK group. GR-induced phosphorylation alterations (p *<* 0.05) were observed in 246 modified sites on 6 kinases. We used Coral (Metz and Deoudes et al.)^25^ to annotate these kinases on a kinome tree (Figure 7A). CaMK2γ, Obscurin, SPEG, and MYLK from the CAMK group had obviously increased phosphorylation trends in E_GM muscles. Conversely, titin, another kinase of the CAMK group, had most phosphorylation sites decreased by GR response. The muscle-specific kinase Obscurin, SPEG, and titin had more than 10 differential phosphorylation sites between the two groups, most of which have not been reported or annotated. So, we mapped these sites to the schematic diagram of their protein domain (Figures 7B–7D). There were GR-induced differential phosphorylation sites near the protein kinase domain of the three muscle-specific kinases. We, therefore, speculate that in addition to CaMK2, the activity of these muscle-specific kinases was also altered in response to GR.

In addition to the above kinases that directly observed differences in phosphorylation, we also predicted kinases that may be involved in the regulation of GR effects based on phosphorylation motif analysis. The peptide sequences of 13 amino acid residues containing phosphorylation sites were subjected to motif enrichment analysis by Motif-X. PxSP and SP were the most enriched sequence patterns in E_GM muscles (Figure 7E), including significantly upregulated phosphorylation sites on PERM1 (S149, S156), OBSCN (S135, S5778, S7721, S8041), etc. RxS was the most enriched motif in E_WT muscles and many phosphorylation sites on TTN belong to this motif (Figure 7E). Based on the assumption that similar protein kinases may regulate similar motifs, we used iGPS1.0 software to predict the phosphokinase upstream of the phosphorylation sites, and then used protein-protein interaction information (PPI) to filter out potential false-positive interactions. In total, we predicted 14,406 regulatory relationships between 246 protein kinases and 1,516 phosphorylation modification sites on 664 proteins identified by the project. Based on our prediction of the regulatory relationship between kinase and phosphorylation sites as a gene set, we used the GSEA method to predict the kinase activity in E_GM and E_WT samples. The normalized enrichment scores obtained by GSEA analysis were considered kinase activity scores (Table S5). The result showed that MAPK family kinase ERK4 and p38d and CDK family kinase CDK5, CDK8, and CDK9, all of which belong to the CMGC kinase group, were significantly enriched in E_GM muscles, while the TKL group RAF family kinases (RAF1, BRAF, and ARAF) were most enriched in E_WT muscles (Figure 7F). This result suggested that these kinases may play a regulatory role in GR-induced phosphorylation alterations in mice’s skeletal muscle.

## Discussion

In this study, GLUT4 p.Q177L mutant mice were obtained using CRISPR/Cas9-mediated gene editing. We choose to target GLUT4 because it is the dominant glucose transporter and an important functional executor of skeletal muscle.^16^^,^^26^ Based on the functional and structural conservation of the glucose transporter family,^17^ we designed a single-amino acid mutant to block substrate binding to the GLUT4 protein, resulting in permanent GR in mice skeletal muscles. As expected, the GLUT4 p.Q177L mutant brought about decreased glucose disposal capacity in *Glut4*^*m*^ mice (Figures 1D and 1E), which were common features of a panel of GLUT4-deficient mice.^27^^,^^28^^,^^29^^,^^30^^,^^31^ *Glut4*^*m*^ mice have normal fasting blood glucose levels probably because *Glut4*^*m*^ restricts glucose uptake in skeletal muscles rather than blocking it completely. *Glut4*^*m*^ mice exhibit elevated expression levels of various glucose transporters, including *Glut4* and *Glut1*, in skeletal muscles. The expression of Glut4 is regulated by the AMPK pathway and MEF2 transcription factors. In our multi-omics datasets, we identified the activation of the AMPK signaling pathway and elevated MEF2 expression in *Glut4*^*m*^ mice. This increase in MEF2 expression potentially enhances Glut4 expression. This compensatory increase partially substitutes for GLUT4’s function in the basal state.

The *Glut4* mutation also affects other tissues (Figure S4). *Glut4* is mainly expressed in skeletal muscles, adipose tissues, and cardiac muscles. *Glut4*^*m*^ restricts glucose uptake in adipose tissues, leading to reduced fat deposition in *Glut4*^*m*^ mice. There were increased cardiac proportion in *Glut4*^*m*^ mice, prompting our investigation into whether this change represents pathological cardiac hypertrophy due to insufficient glucose supply or a physiological compensatory mechanism that may provide enhanced oxygen supply to the more aerobically active skeletal muscles of *Glut4*^*m*^ mice. Furthermore, we observed islet proliferation in the *Glut4*^*m*^ pancreas, which is critical for blood glucose regulation. These adaptations also stabilize blood glucose levels in *Glut4*^*m*^ mice under basal conditions.

The significant features of the *Glut4*^*m*^ mice were enhanced mitochondrial function (Figures 2F–2M) and increased proportion of oxidative slow fibers in skeletal muscles (Figure 3), which persists throughout the life of *Glut4*^*m*^ mice, as shown in transcriptome data at different ages (Figures 2A–2E; Table S2). This was an intriguing result because, typically, insulin-resistance-mediated impairment of GLUT4 function has been reported in obese or T2DM patients, accompanied by more glycolytic fast muscle fibers.^5^ This may be due to the fact that while the GLUT4-deficient mouse models (including *Glut4*^*m*^ and knock-out models) develop insulin resistance with similar outcomes to patients, the mechanism is completely different for the skeletal muscle itself. In the muscles of patients, phosphorylation of PI3K-AKT and AMPK is suppressed due to insulin resistance and metabolic stress,^32^^,^^33^ resulting in blocked GLUT4 vesicle trafficking and decreased GLUT4 expression, as well as declined oxidative phosphorylation metabolism and the oxidative-to-glycolytic fiber type switch.^4^ In other words, GLUT4 deficiency and the loss of oxidative muscle fibers are the consequence of disordered metabolic signaling pathways in the muscles of obese or T2DM patients. In skeletal muscle from *Glut4*^*m*^ mice, insulin signaling was preserved so that AKT could be normally phosphorylated (Figure S2). The GLUT4 mutant limited the rate at which glucose enters muscle cells, thereby activating the cellular energy sensor AMPK (Figures 2C and 6D) for more efficient and thorough utilization of glucose. Based on the above facts, we considered that in the skeletal muscle perspective, the *Glut4*^*m*^ mice could be a more apposite model of GR, rather than insulin resistance.

### GR-induced myokines expression

Previous research has shown that exercise induces skeletal muscle myokine secretion. These myokines participate in regulating glucose and lipid metabolism, mitochondrial function and fiber-type switch of skeletal muscle itself through autocrine or paracrine mechanisms, and mediate the crosstalk between skeletal muscle and other organs by endocrine function.^34^

Apelin encoded by *Apln* is an exerkine that significantly enhances muscle function by triggering mitochondriogenesis, autophagy, and anti-inflammatory pathways in skeletal muscle, as well as enhancing muscle regeneration.^11^ Apelin expression was found to be positively related to physical activity and decreased with age in mice skeletal muscles. The beneficial effects of apelin on skeletal muscle aging are mainly achieved via activation of AMPK, AKT and P70S6K signaling.^11^ Our data confirmed an increased mRNA expression of *Apln* in *Glut4*^*m*^ mice at different ages, suggesting that there might be a positive feedback loop between AMPK activation and apelin production that protects skeletal muscle from atrophy in chronic GR.

Musclin encoded by *Ostn* is another exercise-induced myokine, which enhances physical endurance by promoting PGC-1α-dependent mitochondrial biogenesis.^22^ Serum musclin level is induced by PGC-1α overexpression^35^ and Ca^2+^-dependent Akt activation.^22^ Interestingly, musclin was considered to mainly originate from glycolytic muscle fibers, especially the type IIb fibers.^36^ In patients with type 2 diabetes, obesity or insulin resistance and rodent model fed with a high-fat diet, musclin expressions were increased dominantly due to impaired PI3K-AKT signaling.^37^^,^^38^^,^^39^ However, in *Glut4*^*m*^ mice, musclin mRNA expression was also dramatically increased as the glycolytic fiber ratio decreased, suggesting that musclin expression may not be restricted to glycolytic fibers. In the context of GR, oxidative fibers could also contribute to increased musclin expression, conferring benefits on mitochondrial function.

Apart from apelin and musclin, the mRNA expression of insulin-like growth factor 2 (*Igf2*) and insulin-like growth factor binding protein (*Igfbp5*) were also upregulated in *Glut4*^*m*^ mice regardless of age (Table S3). IGF signaling plays a key role in regulating cell growth and development. *Igf2* is a well-recognized growth-promoting factor involved in the positive regulation of muscle mass and muscle mitochondrial biogenesis.^40^^,^^41^ With age, the expression of *Igf2* decreased dramatically. Here in *Glut4*^*m*^ mice, the higher expression of *Igf2* might help skeletal muscle maintain a youthful phenotype. Moreover, a previous study suggested that upregulated expression of *Igfbp5* in muscle satellite cells during aging might be a compensatory mechanism to improve muscle regeneration.^42^ Our data agree with this view and suppose that skeletal muscle GR increases the expression of *Igf2* and *Igfbp5*, thus preventing aging. In addition, the vascular endothelial growth factors *Vegfa* and *Vegfb* and angiopoietin family member *Angpt1* were expressed at higher mRNA levels in *Glut4*^*m*^ mice than in WT mice at all ages (Figure 4D; Table S3). These molecules secreted by skeletal muscle promote angiogenesis and were proven to play key roles in slow fiber formation.^43^ We suppose that the increased angiogenesis in *Glut4*^*m*^ muscle increases oxygen and glucose supply to skeletal muscles, which might compensate for GR and these changes were beneficial for anti-ageing processes and made skeletal muscle preserve “young” transcriptome signatures in *Glut4*^*m*^ mice. This piece of work sheds new light on inducing beneficial myokine secretion, improving skeletal muscle metabolism and aging.

### The cooperation of AMPK and CaMK2 signaling in *Glut4*^*m*^ muscle

Through the LIT experiments, we compared the effects of low-intensity exercise and GR on skeletal muscle proteome, respectively. After LIT, the oxidative phosphorylation of skeletal muscle in WT mice was enhanced, but there was no change in their muscle fiber type. However, LIT brought dramatic influences on the skeletal muscle phenotype of *Glut4*^*m*^ mice, including fast-to-slow fiber type switch and activation of cAMP signaling and calcium signaling pathways (Figure 5F). These changes suggest that GR may increase the benefits of low-intensity exercise for skeletal muscle.

Phosphorylation is one of the most important post-translational modifications in regulating biological processes. With the development of high-throughput protein sequencing technology, recently, skeletal muscle phosphoproteomes in different states have been widely reported. Hoffman et al.^44^ reported the global changes in skeletal muscle phosphoproteome after acute exercise and revealed the interaction between multiple kinases with AMPK substrates. Potts et al.^45^ described the phosphorylation changes of skeletal muscle after a bout of maximal-intensity contraction and found that phosphorylation of the Z-disk kinases SPEG and Obscurin were significantly downregulated in response to high-intensity contraction. In the present work, we have a similar finding that the phosphorylation state on SPEG, OBSCN, SYNPO2, and SYNPO2L in the Z-disk significantly differed between *Glut4*^*m*^ mice and WT mice. In contrast, GR in *Glut4*^*m*^ muscles led to markable increased phosphorylation on these kinases (Figure 6F). Interestingly, these kinases that had the most significant phosphorylation differences all belong to the CAMK kinase family (Figure 7A). The important CAMK family member CaMK2γ, which regulates slow fiber development, was also significantly activated in *Glut4*^*m*^ mice, mediating the increase of slow muscle fibers through downstream transcription factor MEF2 (Figure 6G).

Our results confirm the important role of CaMK2 signaling in regulating skeletal muscle flexibility and hypothesized that GR may somehow affect the activation of CaMK2. Combining our data with existing reports,^46^ we suggest that phosphorylation of PERM1 most likely coordinates CaMK2-MEF2 signaling and AMPK-PGC-1α signaling to synergistically promote slow fiber development and mitochondrial production in skeletal muscles, providing potential therapeutic targets for obesity-related metabolic diseases.

### Comprehensive molecular features of skeletal muscle under GR

This study comprehensively delineates the molecular characteristics of skeletal muscle under GR at the levels of gene transcription, protein translation, and post-translational modifications. In *Glut4*^*m*^ mice, the increase in MHY7^+^ fibers is revealed to be more than a mere elevation in MYH7 expression. Various molecules associated with the physiological functions of slow-twitch fibers exhibit corresponding changes. For instance, the expression of muscle-specific sarcoplasmic reticulum calcium pump ATP2A2, muscle calcium-binding proteins (TNNC1, TNNI1, TNNT1), and internal calcium store (CASQ2) is elevated. Slow-twitch fibers, primarily involved in aerobic metabolism, show enhanced expression or selective phosphorylation/dephosphorylation of enzymes related to glucose aerobic oxidation and the TCA cycle. This facilitates the thorough breakdown of substrates like glucose for energy supply, reflecting the metabolic adaptation of skeletal muscles under GR conditions. These molecular changes are regulated by the activation of the AMPK and CaMK2 pathways. Additionally, we identified several GR-related myokines through transcriptomic analysis and observed different phosphorylation modifications in other signaling pathways or enzymes under GR conditions through phosphoproteomic analysis. The mutual validation of these data across multiple regulatory levels provides robust foundational support for target selection aimed at enhancing skeletal muscle aerobic metabolism under GR.

### Conclusion

This study constructed a GLUT4-mutated mouse model for skeletal muscle GR. In the absence of exercise intervention, GR promotes the formation of slow-twitch fibers during daily activities, enhances mitochondrial function, and promotes myokine expressions during lifespan. Exerkines such as musclin and apelin were found upregulated at mRNA level in gastrocnemius from *Glut4*^*m*^ mice. Through bioinformatics mining, we confirmed the dominant roles of AMPK and CaMK2 in skeletal muscle flexibility and speculated that PERM1 is a potential drug target for improving muscle metabolic health. These results constitute a multi-omics database containing the molecular information in skeletal muscle at different ages, substrate supply, and exercise stimuli, providing data support for skeletal muscle physiology, metabolism, and drug development. We demonstrated that skeletal muscle glucose restriction may synergistically enhance the beneficial effects of low-intensity exercise. The comprehensive dataset not only provides many new potential targets for the development of drugs for metabolic diseases but also offers novel ideas for treatment options in lifestyle interventions.

### Limitations of the study

This study based on the GR mouse model, provided a comprehensive observation of multi-level molecular characteristics in skeletal muscle under GR conditions. High-throughput omics research offers valuable datasets for rapid screening of impactful molecules or pathways on phenotypes. However, detailed mechanistic investigations are ongoing. For instance, the heightened phosphorylation of Perm1, a molecule potentially coordinating the AMPK and CaMK2 pathways, might help skeletal muscle adaptation to GR. Future studies will focus on verifying if Perm1 phosphorylation independently activates AMPK and CaMK2 signaling, thus promoting slow muscle fiber development. It is also necessary to explore the specific modification sites and corresponding kinases that play a key role. Moreover, the effects of *Glut4* mutations in mice are systemic. When skeletal muscle glucose uptake is limited, compensatory changes in vital tissues like fat, liver, and pancreas, crucial for blood sugar regulation, warrant exploration. Identifying key pathways in this compensation is a priority. Additionally, the increased heart weight in *Glu4*^*m*^ mice raises questions: is it due to pathological myocardial hypertrophy from *Glut4* mutation or a physiological adaptation to enhance oxygen demand? Further investigations are needed to unravel these complexities.

## STAR★Methods

### Key resources table

**Table:** 

REAGENT or RESOURCE	SOURCE	IDENTIFIER
**Antibodies**		

Anti-Glucose Transporter GLUT4 antibody	Abcam	Cat#ab654; RRID:AB_305554
Anti-Glucose Transporter GLUT1 antibody [EPR3915]	Abcam	Cat#ab115730; RRID:AB_10903230
COX IV (4D11-B3-E8) Mouse mAb	Cell Signaling Technology	Cat#11967; RRID:AB_2797784
SCD1 (C12H5) Rabbit mAb	Cell Signaling Technology	Cat#2794; RRID:AB_2183099
Phospho-AMPKα (Thr172) (40H9) Rabbit mAb	Cell Signaling Technology	Cat#2535; RRID:AB_331250
AMPKα Antibody	Cell Signaling Technology	Cat#2532; RRID:AB_330331
Anti-CaMK2 beta gamma delta (phospho T287) antibody	Abcam	Cat#ab182647
Anti-MEF2A + MEF2C antibody [EPR19089-34]	Abcam	Cat# ab197070; RRID:AB_2629454
Phospho-Akt (Ser473) (D9E) XP® Rabbit mAb	Cell Signaling Technology	Cat#4060; RRID:AB_2315049
Akt (pan) (C67E7) Rabbit mAb	Cell Signaling Technology	Cat#4691; RRID:AB_915783
GAPDH (14C10) Rabbit mAb	Cell Signaling Technology	Cat#2118; RRID:AB_561053
Anti-Slow Skeletal Myosin Heavy chain antibody [NOQ7.5.4D]	Abcam	Cat#ab11083: RRID:AB_297734
Anti-Fast Myosin Skeletal Heavy chain antibody	Abcam	Cat#ab91506: RRID:AB_10714690
Anti-rabbit IgG, HRP-linked Antibody	Cell Signaling Technology	Cat#7074; RRID:AB_2099233
Anti-mouse IgG HRP-Linked secondary antibody	Yangguangyingrui	Cat#SBA0002
Goat Anti-Mouse IgG H&L (Alexa Fluor® 594)	Abcam	Cat#ab150116; RRID:AB_2650601
Donkey Anti-Rabbit IgG H&L (Alexa Fluor® 488)	Abcam	Cat#ab150073; RRID:AB_2636877

**Chemicals, peptides, and recombinant proteins**		

D-(+)-Glucose	Sigma-Aldrich	Cat#G7528
Insulin solution from bovine pancreas	Sigma-Aldrich	Cat# I0516

**Critical commercial assays**		

TRIzol™ Plus RNA Purification Kit	Invitrogen™	Cat#12183555

**Deposited data**		

Raw data of RNAseq	This paper	SRA: PRJNA924706
Raw data of proteomics	This paper	PX: PXD040132
Raw data of phosphoproteomics	This paper	PX: PXD040294

**Experimental models: Organisms/strains**		

Mouse: C57BL/6J	Beijing HFK Bioscience Co., LTD	http://www.hfkbio.com
Mouse: Glut4^m^: C57BL/6J	This paper	N/A

**Oligonucleotides**		

*m*-slc2a4-gRNA: 5′-TAGGCCATC GTCATTGGCATTC-3′	This paper	N/A
Primer-F: 5′-TTGGACGGTTCCT CATTGGC-3′	This paper	N/A
Primer-R: 5′-GCTCTTCCTTCCA GCCCAAAT-3′	This paper	N/A
Primers for qPCR, see Table S7	This paper	N/A

**Software and algorithms**		

ImageJ software	Schneider et al.^47^	https://imagej.nih.gov/ij/
DESeq2 software	Love MI et al.^48^	https://www.bioconductor.org/packages/release/bioc/html/DESeq2.html
MaxQuant software	Tyanova S et al.^49^	http://www.maxquant.org
MoMo software	Cheng A et al.^51^	http://meme-suite.org
Omicsmart platform	Gene *Denovo* Biotechnology Co., Ltd	http://www.omicsmart.com
Cytoscape software	Shannon P et al.^52^	https://cytoscape.org

### Resource availability

#### Lead contact

Further information and requests for resources and reagents should be directed to and will be fulfilled by the lead contact, Shulin Yang (yangshulin@caas.cn).

#### Materials availability

The *Glut4*^*m*^ mouse model constructed in this experiment are maintained at IAS, CAAS. All biological samples are available on a reasonable request from the lead contact, Shulin Yang (yangshulin@caas.cn).

#### Data and code availability

• RNA-sequencing data generated in this study have been deposited in Sequence Read Archive (SRA) (https://trace.ncbi.nlm.nih.gov/Traces/sra/) under the accession number PRJNA924706. The proteomics and phosphoproteomics data have been deposited to the ProteomeXchange Consortium (http://proteomecentral.proteomexchange.org) via the iProX partner repository^53^^,^^54^ with the dataset identifier PXD040132 and PXD040294. All omics datasets are publicly accessible. All original experimental data generated in this study will be shared by the lead contact upon request.
• No original code is reported in this study.
• Any additional information required to reanalyse the data reported in this paper is available from the lead contact upon request.

### Experimental model and study participant details

#### Animals and ethics statement

WT C57BL/6J mice were purchased from Beijing HFK Bioscience Co., LTD (Beijing, China). *Glut4*^*m*^ mice were generated by the Institute of Laboratory Animal Sciences, Chinese Academy of Medical Sciences. To avoid interference with normal GLUT4 proteins, only mice with double allele *slc2a4* mutation (*Glut4*^*m*^ homozygous) were used in this experiment. Male mice at the age of 12–60 weeks were used in this study. Mice were fed with a regular laboratory mouse diet (20% protein, 4% fat, 66% carbohydrate) and water *ad libitum*. Animal breeding, management, and experiments were entrusted to Beijing HFK Bioscience Co., LTD (Beijing, China) and all animal studies were approved by the Institutional Animal Care and Use Committee, permit No. IACUC-20181113. All animals were treated humanely following the Guide for the Care and Use of Laboratory Animals (National AcademyPress Washington D.C. 1996).

### Method details

#### Genetic editing of mice

In brief, *Glut4*^*m*^ mice were constructed using CRISPR/Cas9 system targeting the exon 5 of the C57BL/6J mice *slc2a4* gene. SgRNAs were designed using an online tool (crispr.mit.edu/job/7002675348910624) and the sgRNA with the highest efficiency (*m*-slc2a4-gRNA 5′-TAGGCCATCGTCATTGGCATTC-3′) was selected. A 90 bp oligo DNA with the A→T mutant site was synthesized as the homologous recombination repair (HDR) template (Figure S1). The Cas9 protein, sgRNA, and HDR template were microinjected into C57BL/6J mice zygotes, which were consequently transplanted into pseudo-pregnant mothers. Genotyping of *Glut4*^*m*^ and WT mice was performed by PCR amplification and gene sequencing of a 340 bp product which includes the target site (Primer-F: 5′-TTGGACGGTTCCTCATTGGC-3′, Primer-R: 5′-GCTCTTCCTTCCAGCCCAAAT-3′, Figure S1).

#### GTT and ITT

GTT and ITT experiments were performed on male mice at 20 weeks (WT = 8, *Glut4*^*m*^ = 6). Before the experiments, fasted glucose levels were tested. Mice fasted for 16 h and received i.p. glucose at 2 g/kg of body weight in GTT, or fasted for 8 h and received i.p. insulin at 1 U/kg of body weight in ITT. One drop of blood was sampled from the tail and whole blood glucose was measured by a glucometer (Ultra OneTouch, Johnson, America).

#### Body composition

Body composition was measured using the nuclear magnetic resonance (NMR) method. Age-matched male mice (n = 5 per group) were measured. Before NMR measurement, mice fasted 8 h and the reference body weights were measured using an electronic scale. Mice were fixed in a cylindrical tube for measurements and each mouse was measured 3 times. Data including fat mass, lean mass, and body water were collected for statistical analysis.

#### Indirect calorimetry

An indirect calorimetry test was carried out on 12-week-age male mice (n = 10 for *Glut4*^*m*^ and n = 8 for WT) using an open-circuit calorimetry system (Oxymax, Columbus). Each mouse was housed individually in a calorimeter chamber at 25°C, with *ad libitum* water and a 12-h photoperiod. The air flow (L/min) into the box is controlled by the air valve and O_2_% and CO_2_% were recorded. An air sample was taken from the box every 9 min and O_2_% and CO_2_% were measured. These data were used to automatically calculate mice’s oxygen consumption (VO_2_, mL/h/kg), carbon dioxide production (VCO_2_, mL/h/kg), and RER (VCO_2_/VO_2_). Data collected in the first 2 h were not used in the analysis in order to balance and acclimatize mice to the environment in the calorimeter chamber. Data were collected continuously for the next 24 h for the indirect calorimetry analysis.

#### Western blotting

Total proteins were extracted from 100 mg of each gastrocnemius muscle samples (n = 4 per group) using Tissue Protein Extraction Reagent (Thermo #78510). Protease inhibitors (Roche 04693159001) and phosphatase inhibitors (Roche 04906837001) were used to prevent protein degradation and dephosphorylation. Protein samples were mixed with loading buffer containing SDS and β-mercaptoethanol and denatured by boiling at 100°C. 20 μg of denatured total protein were loaded into each line. Primary antibodies recognizing GLUT4 (Abcam ab654), GLUT1 (Abcam ab115730), COX IV (CST #11967), SCD-1(CST #2794), phospho-AMPK (CST #2535), total AMPK (CST #2532), phospho-CaMKII (Abcam ab182647), MEF2 (Abcam ab197070), phospho-AKT (CST #4060) and pan-AKT (CST #4691) were used to detect the relative protein expressions and GAPDH (CST #2118) were used as house-keeper. Anti-rabbit IgG secondary antibody (CST #7074) and anti-mouse IgG secondary antibody (SBA0002) were used respectively. Blots were imaged by Tanon 5200 Automated Chemiluminescence Image Analysis System (Tanon, Shanghai, China). Each test was repeated 3 times. The grayscale density was analyzed using ImageJ software. Statistical analysis was performed by Prism8 using the Student’s *t* test.

#### Immunofluorescence staining and quantification of myofibrils

Gastrocnemius muscle samples were fixed in 4% paraformaldehyde, embedded with paraffin, and sliced into 4 μm sections. Paraffin sections were dewaxed in xylene and rehydrated in graded alcohol. Sections were microwave-heated to 95°C and kept for 20 min in Tris-EDTA (pH 9.0) buffer for the antigen retrieval step. Incubate sections in 5% BSA, 5% goat serum, 5% donkey serum in PBST at room temperature for 1 h to block unspecific binding of the antibodies. Antibodies were used as follows: MYH7 (Abcam, ab11083) and MYH4 (Abcam, ab91506) as the primary antibody; Goat Anti-Mouse IgG H&L (Alexa Fluor 594) (ab150116) and Donkey Anti-Rabbit IgG H&L (Alexa Fluor 488) (ab150073) as the secondary antibody. Fluorescent images were taken with ECLIPSE Ni-U fluorescent microscope (Nikon, Japan) with Digital Sight 10 imaging system and NIS-Elements software. The number and area of muscle fibers in each image were counted using ImageJ software.^47^

#### Transmission electron microscopy and quantification of mitochondria

Gastrocnemius medial head and soleus were dissected and fixed with 2.5% glutaraldehyde sample fixative for electron microscopy. Sample processing and frozen ultra-thin section preparation were performed by Institute of Food Science and Technology, CAAS. Ultra-micrographs at 7000-30000× magnificence was acquired using H-7500 transmission electron microscope (HITACH, Japan). The number and area of mitochondria in the images were counted using ImageJ software.

#### RNA sequencing and data analysis

RNA-seq samples were collected from male WT and *Glut4*^*m*^ mice at 12-, 24-, 36- and 60-week-old. There were 32 mice (16 WT and 16 *Glut4*^*m*^, n = 4 per growth stage) used in the transcriptome study. Total RNA of mouse gastrocnemius muscle samples was extracted using Trizol reagent kit (Invitrogen, USA) according to the manufacturer’s protocol. cDNA library was constructed and sequenced using Illumina Novaseq6000 by Gene *Denovo* Biotechnology Co. (Guangzhou, China). Clean reads were filtered by fastp (version 0.18.0), mapped to mouse reference genome (GRCm38 Ensembl release100), and calculated to FPKM (fragment per kilobase of transcript per million mapped reads) value by HISAT2.2.4 to quantify the expression of each transcript. Differentially expressed genes (DEGs) were analyzed using DESeq2 software^48^ and genes with p value < 0.05 and absolute fold change> 1.5 were considered as DEGs.

#### Low-intensity treadmill training (LIT)

For the LIT training group, WT and *Glut4*^*m*^ mice (n = 8 per group) were trained 30 min every day on a treadmill at slow-to-moderate speed (8–10 m/min gradually) (Figure 5A). LIT training lasted 4 weeks, from 8-week-age to 12-week-age and there were 6 training days with 1 resting day every week. 12-week-age WT and *Glut4*^*m*^ mice (n = 8 per group) without additional training were considered as control group.

#### Proteomics

Gastrocnemius muscle samples used in proteomics analysis were collected from 12-week-old WT and *Glut4*^*m*^ mice, with or without LIT training (n = 4 per group). For untrained group (NE_WT and NE_GM), samples were collected as described above. For LIT group (E_WT and E_GM), samples were collected immediately after the last training and preserved in liquid nitrogen. One-half of each gastrocnemius muscle was used in MS-based proteome analysis. Proteins were extracted with lysis buffer (8 M urea, 1% Protease Inhibitor Cocktail, 1% Phosphatase Inhibitor) and the protein concentration was determined with BCA kit according to the manufacturer’s instructions. Protein solution was reduced with 5mM dithiothreitol for 30 min at 56°C and alkylated with 11mM iodoacetamide for 15 min at RT in darkness, then diluted by 100mM TEAB, and digested by trypsin. Digested peptides were desalted by Strata X C18 SPE column (Phenomenex), vacuum-dried and reconstituted in 0.5 M TEAB for TMT labeling (Thermo Scientific). Labeled peptides were separated with a gradient of 8%–32% acetonitrile (pH 9.0) to 14 fractions by high pH reverse-phase HPLC using Aglient 300Extend C18 column (5 μm particles, 4.6 mm ID, 250 mm length) on an EASY-nLC 1000 UPLC system. Tandem mass spectrometry (MS/MS) was performed using the Q ExactiveTM Plus system (Thermo Scientific) coupled online to the UPLC. MS/MS data were processed by MaxQuant (v.1.5.2.8)^49^ and searched against UniProt *Mus musculus* database (https://www.uniprot.org/peptide-search, Mus_musculus_10090, size: 17045 entries). The false discovery rate was adjusted to <1% at the peptide and protein levels.

#### Phosphoproteomics

Protein was extracted from the rest half of gastrocnemius muscles from LIT-trained WT and *Glut4*^*m*^ mice (E_WT and E_GM, n = 4 per group). Phosphoproteomics samples were prepared as described previously.^50^ Phosphopeptide was enriched using titanium dioxide (TiO2) beads. Tmt-labelled phosphopeptides were separated by high pH reverse-phase HPLC to 8 fractions and then subjected to MS analysis. The motif characteristics of the phosphorylation modification sites were analyzed based on the motif-x algorithm using MoMo software.^51^ All identified phosphorylation sites with 6 upstream and 6 downstream amino acids were analyzed. A certain peptide sequence that counted more than 20 times with a p value < 0.000001 was considered a motif of phosphorylation modification.

### Quantification and statistical analysis

Data were analyzed by unpaired Student’s *t* test and showed as mean ± standard deviation. p-value < 0.5 was considered statistically significant and p value < 0.01 was considered a very significant difference. An individual mouse was counted as a biological replicate, and all experiments were replaced at least twice. GraphPad Prism 8.0 were used in statistical analysis and data visualization. For omics studies, bioinformatic analysis and visualization (including PCA, Venn map, heatmap, KEGG enrichment, GO enrichment, GSEA and trend analysis) were performed using Omicsmart, a real-time interactive online platform for data analysis (http://www.omicsmart.com) and R (version 3.6.1). For gene-protein-phosphorylation modification network analysis, gene/protein ID was conversed to ENSG using g: Profiler (https://biit.cs.ut.ee/gprofiler/convert), protein interactions were searched against the STRING database and the interaction networks from STRING were visualized in Cytoscape (v3.6.1).^52^

## Supplemental information

Document S1. Figures S1–S17 and Tables S2, S3, S6, and S7

Table S1. GO and KEGG enrichment of the 52 GR genes, related to Figure 2

Table S4. KEGG enrichment of differentially expressed proteins, related to Figure 5

Table S5. Quantified phosphorylation sites and kinase activity prediction, related to Figures 6 and 7
