# Supplementary material for: Glucose restriction enhances oxidative fiber formation: A multi-omic signal network involving AMPK and CaMK2
Source: iScience. 2023 Nov 29;27(1):108590. doi: 10.1016/j.isci.2023.108590 (PMC10755363; doi:10.1016/j.isci.2023.108590)
Supplement: Document S1. Figures S1–S17 and Tables S2, S3, S6, and S7 [file mmc1.pdf]

iScience, Volume *27*

Supplemental information

Glucose restriction enhances oxidative fiber formation: A multi-omic signal network involving AMPK and CaMK2

Kaiyi Zhang, Ning Xie, Huaqiong Ye, Jiakun Miao, Boce Xia, Yu Yang, Huanqi Peng, Shuang Xu, Tianwen Wu, Cong Tao, Jinxue Ruan, Yanfang Wang, and Shulin Yang

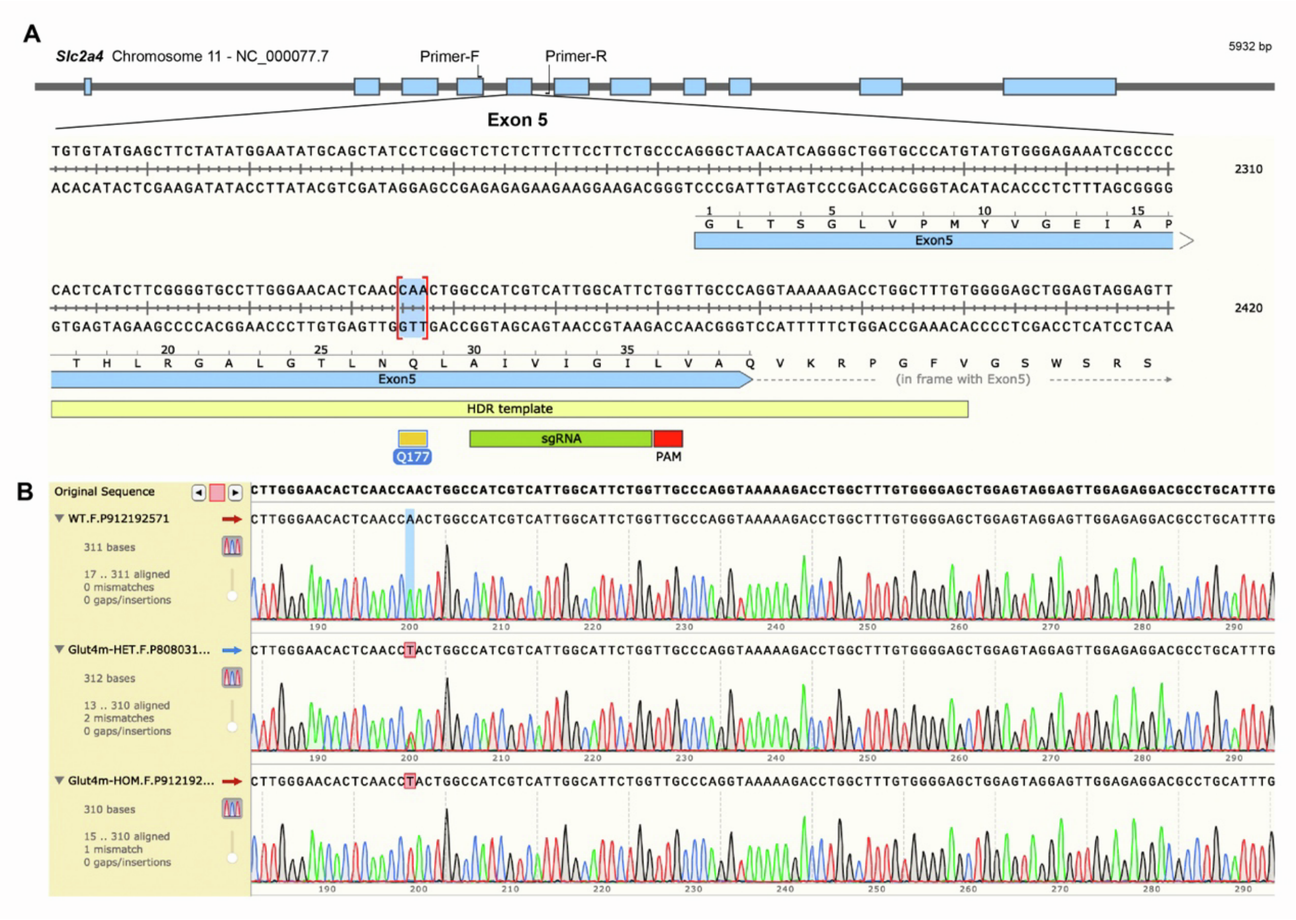

Figure S1 Information of gene editing, related to Figure 1

(**A**) Schematic diagram of mouse *Slc2a4* gene. The CAA codon encoding the 177th glutamine residue (Q177) is located in the fifth exon. sgRNA with the highest efficiency was selected and a 90 bp homologous recombination repair (HDR) template was designed according to the genetic editing target and CRISPR/Cas9 cutting site. A pair of PCR primers was designed to amplify a 340 bp product containing the target sequence for genotyping. (**B**) Sequencing results of the genotyping PCR products. The CAA codon encoding Q177 was replaced with CTA (CUA codon) encoding L177. An overlapping peak of A and T was detected in Glut4^{m} heterozygous (HET) and the A peak was completely replaced by a T peak in Glut4^{m} homologous (HOM).

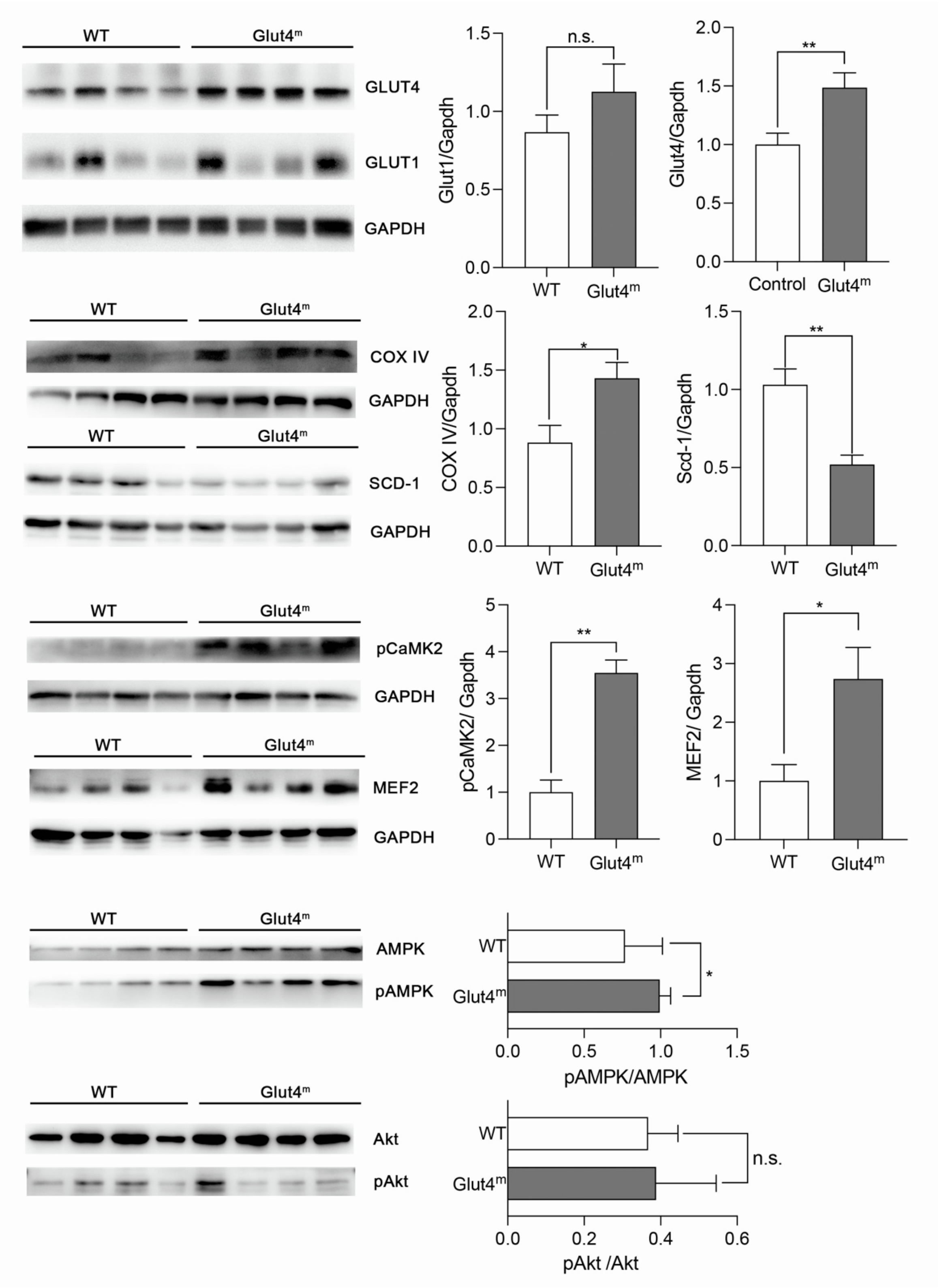

Figure S2 Statistics of immunoblotting results, related to Figure 1, 2 and 6

The grayscale density of blots was analyzed using ImageJ software. Statistical analysis was performed by Prism8 using the Student's t-test. Data are represented as mean± SEM. * $p< 0.05$, ** $p< 0.01$, n.s. not significant.

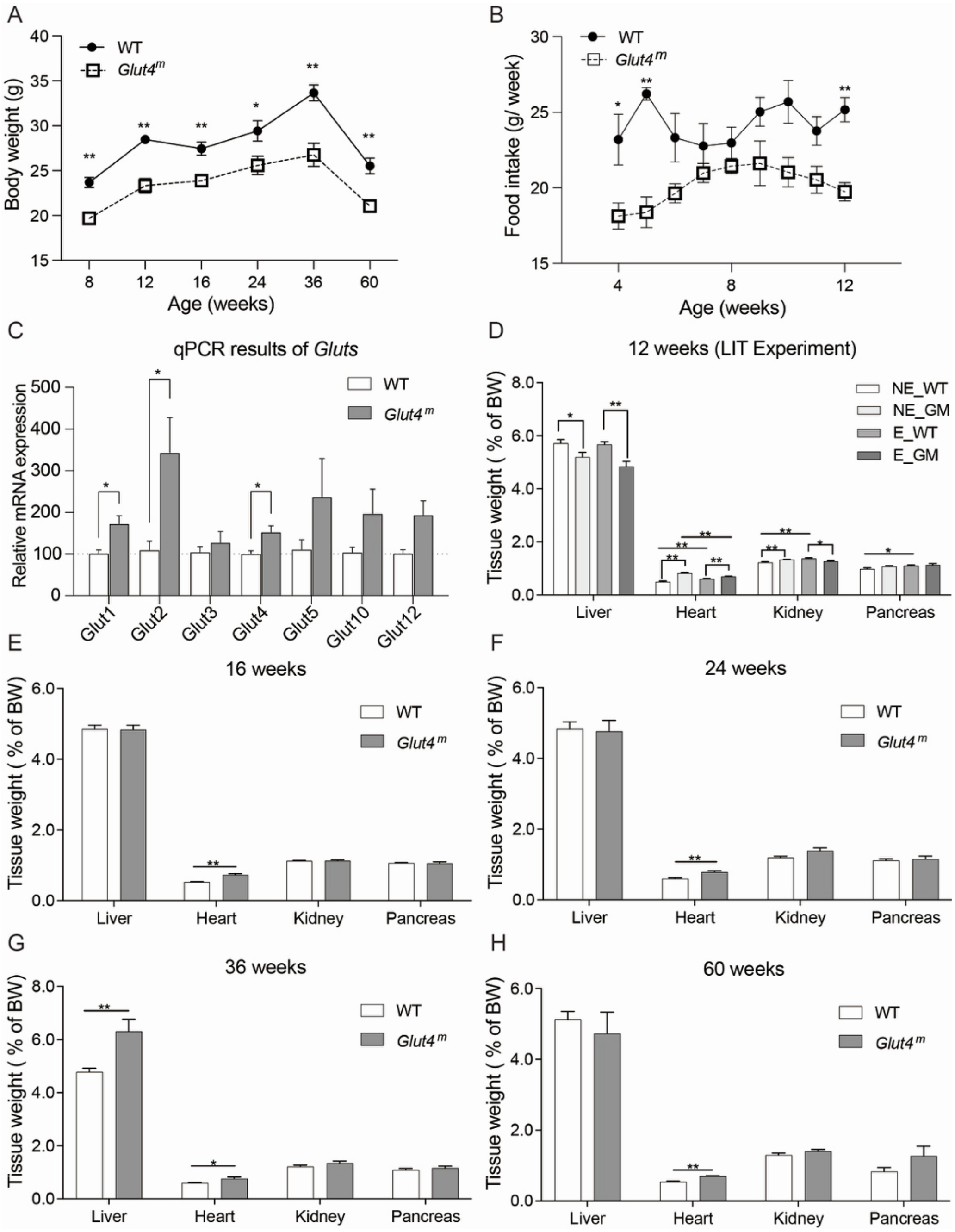

Figure S3 qPCR results of Gluts expression and the global impact of Glut4 mutation, related to Figure 1

(**A**) Body weight of *Glut4*^{m} and WT mice at 8-60 weeks. (**B**) Food intake of *Glut4*^{m} and WT mice at 4-12 weeks. The weekly food intake data for mice were recorded per cage (5 mice per cage), and the average weekly intake per mouse was calculated. Each group consisted of 3 cages (15 mice in total) for analysis. (**C**) Relative mRNA expression of Gluts in mice skeletal muscle. Data were calculated by 2^ddCT method (n=3 per group). (**D**) The percentage of tissue weights to the body weights of 12-week male mice in the low-intensity treadmill (LIT) training experiments. NE_WT and NE_GM, WT and *Glut4*^{m} mice without LIT training; E_WT and E_GM, WT and *Glut4*^{m} mice with 4-week LIT training. (**E-H**) The percentage of tissue weights to the body weights of 16-, 24-, 36- and 60-week mice. Statistical analysis was performed by Prism8 using the Student's t-test. Data are represented as mean± SEM. * p< 0.05, ** p< 0.01.

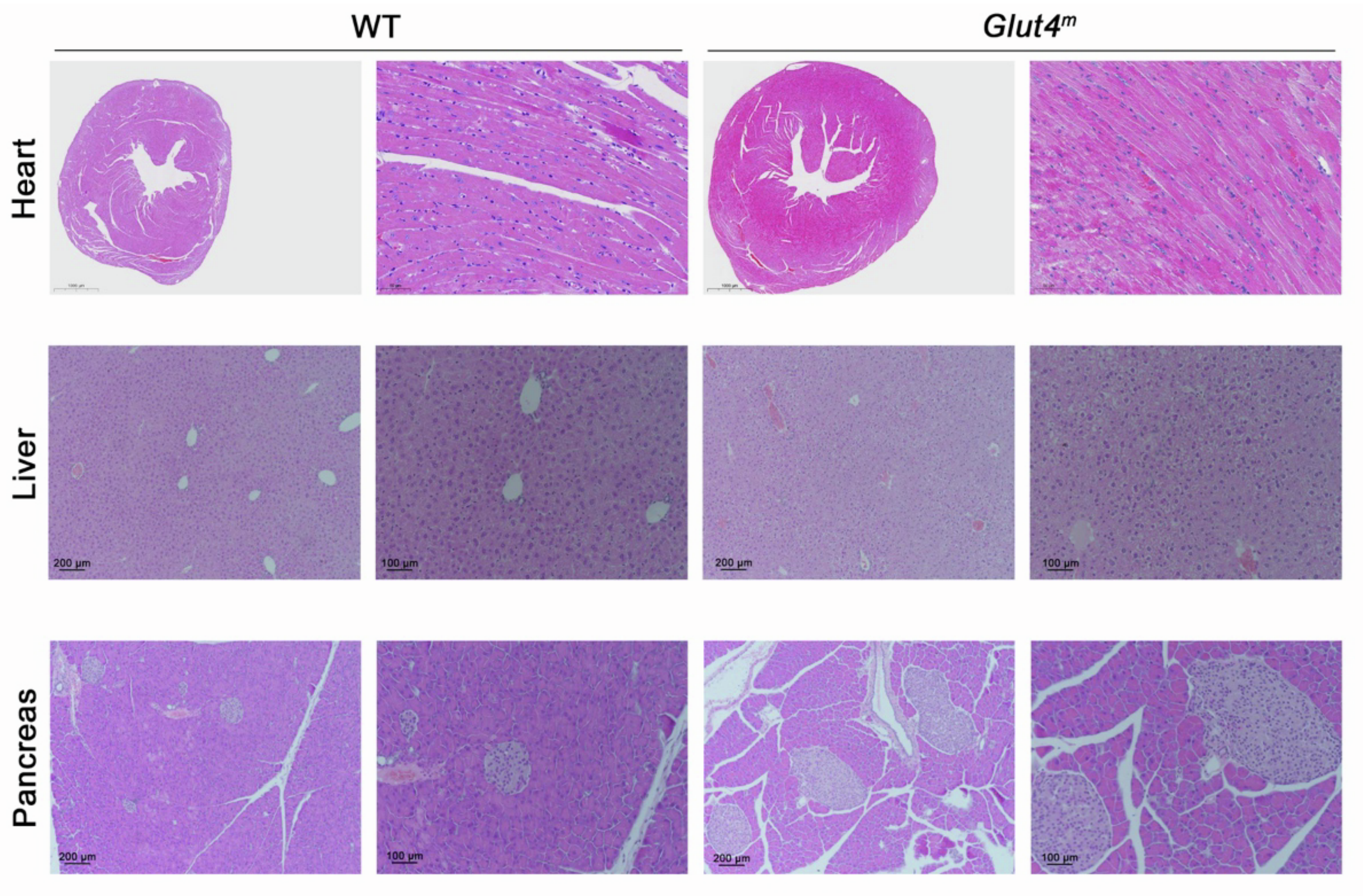

Figure S4 Histological results of other organs, related to Figure 1

HE staining of the heart, liver and pancreas samples from WT and *Glut4*^{m} mice. Scale bars are marked on the images.

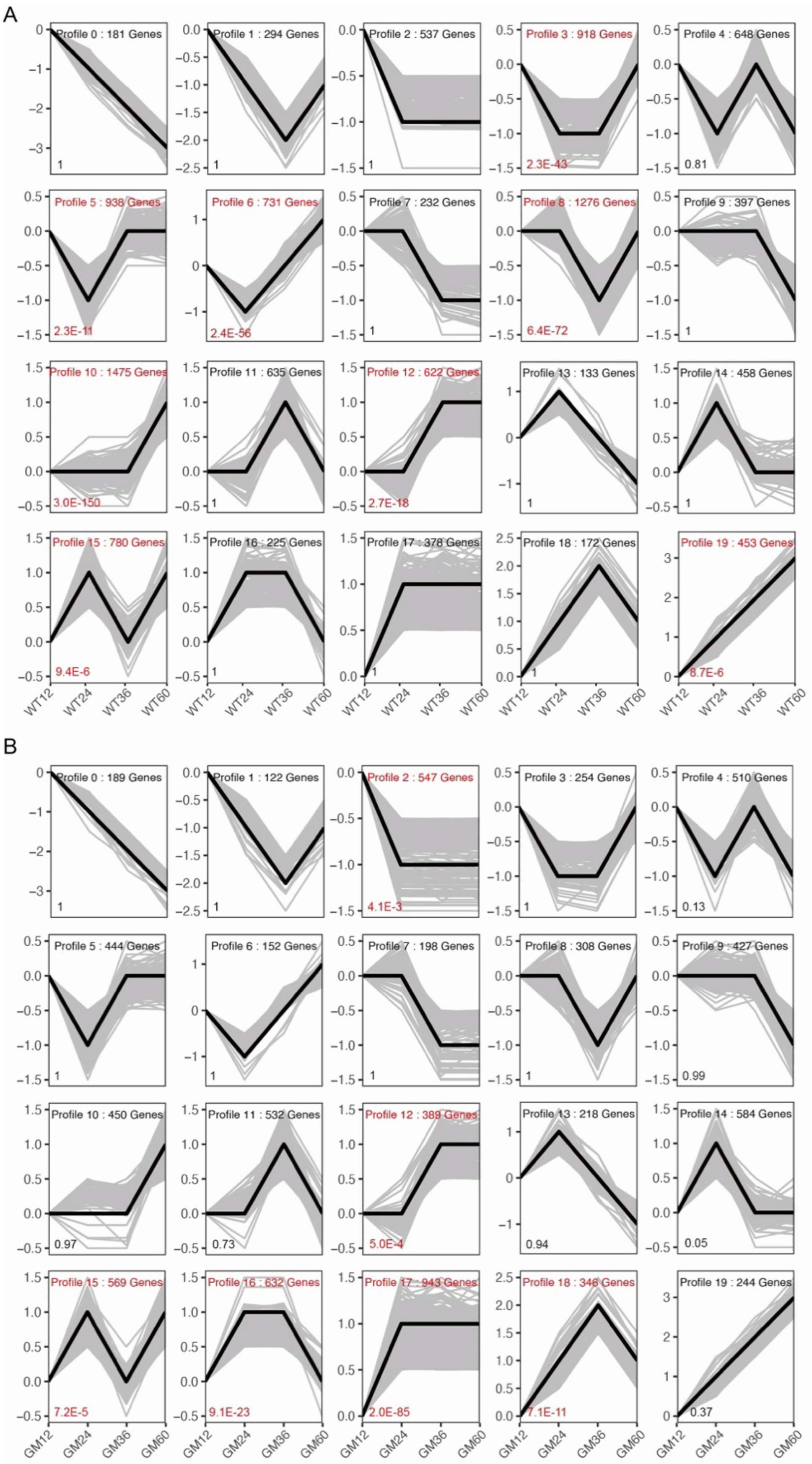

Figure S5 Overview of series test of clusters of the transcriptome data, related to Figure 4

Series test of age-related gene expression in WT samples (**A**) and in *Glut4*^{m} samples (**B**). The expression patterns of the four growth stages (12-, 24-, 36- and 60-week age) were clustered into 20 profiles (profile 0-19). The number of genes in each profile was annotated on the top of the shadow map and the *p*-value of each profile (indicating the number of genes) was marked at the bottom.

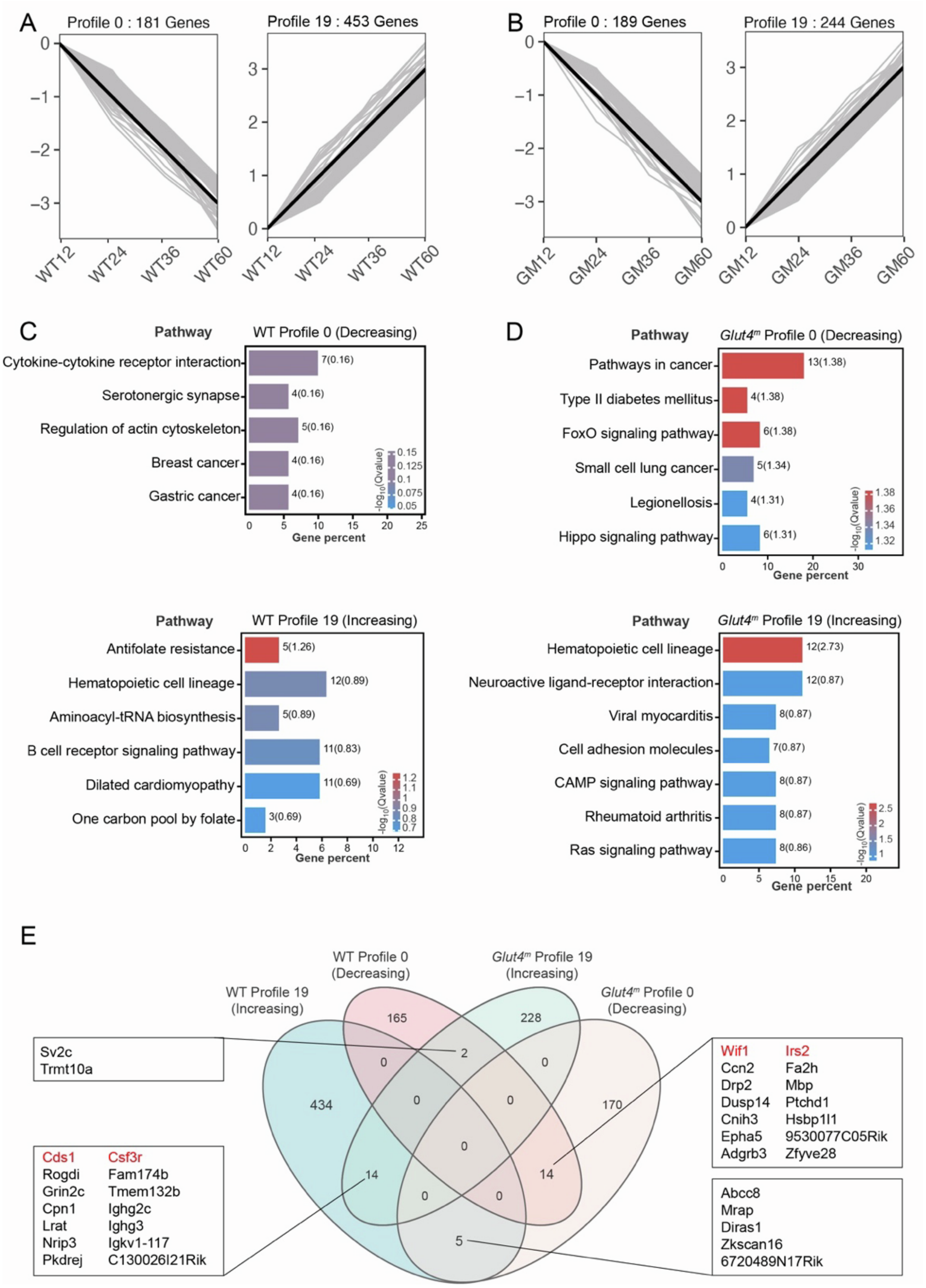

Figure S6 Age-dependent gene expression patterns in mice skeletal muscle, related to Figure4

(**A-B**) The shadow map of profile 0 (genes with declined expression with age) and profile 19 (genes with increased expression with age) in WT mice (**A**) and *Glut4^{m}* mice (**B**). The grey line indicates a single gene expression and the black line shows the general trend. (**C-D**) KEGG enrichment of the age-related genes (profile 0 and profile 19) in WT mice (**C**) and in *Glut4^{m}* mice (**D**). The number of enriched genes and the gene percentage in each pathway were labeled next to the bar. (**E**) The overlapping age-related genes in WT and *Glut4^{m}* mice.

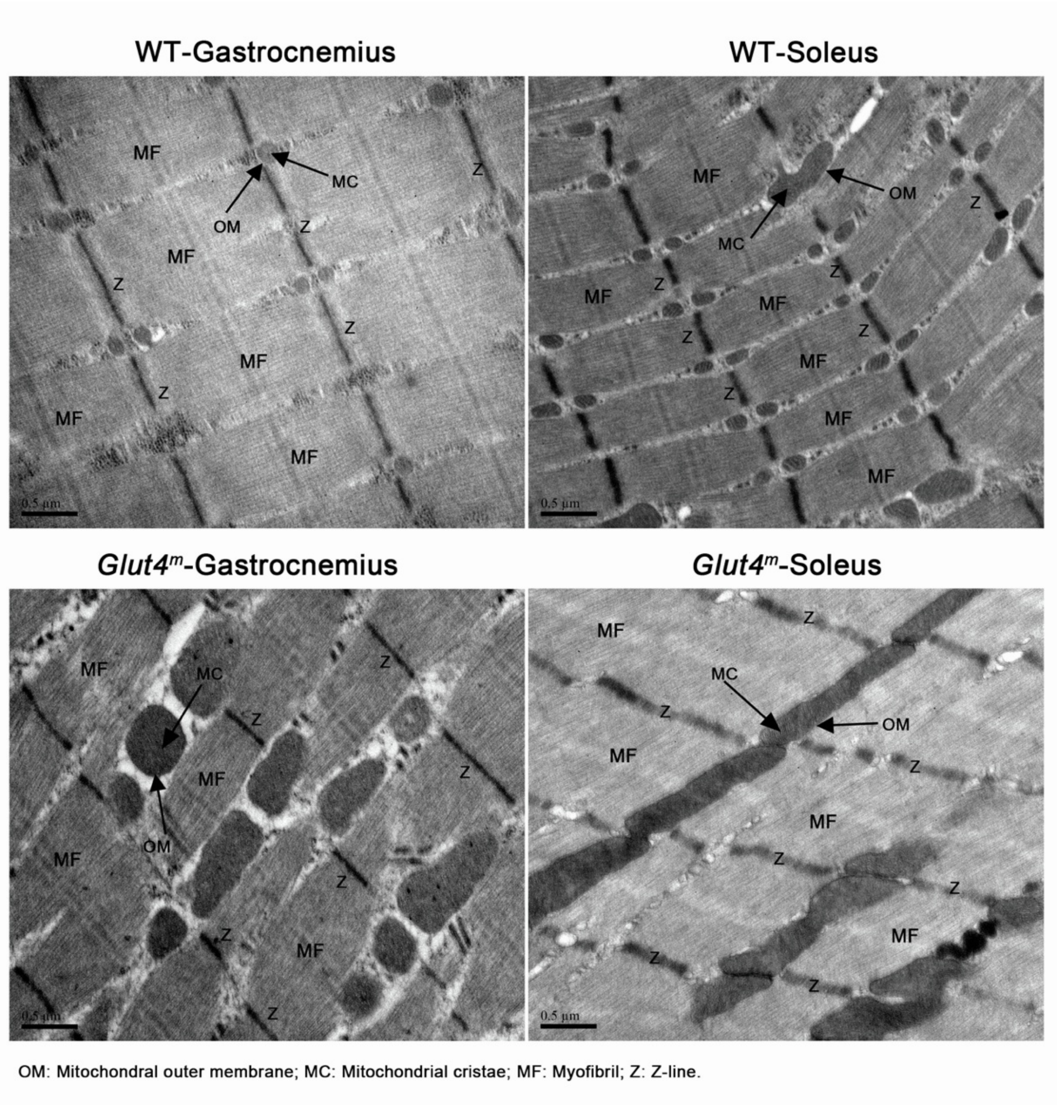

Figure S7 Representative transmission electron microscope images of gastrocnemius and soleus from *Glut4^m^* and WT mice, related to Figure 2

Magnification: 30000×. Scale bar: 0.5 μm.

Table S2 GSEA enriched KEGG pathways of 4 age groups, related to Figure 2

KEGG pathway	Size	ES	NES	p-value	FDR
**12 weeks**					
Arginine biosynthesis	20	0.71	1.94	0	0.0202
Propanoate metabolism	34	0.61	1.92	0	0.0114
Valine, leucine and isoleucine degradation	57	0.52	1.76	0	0.0308
Ribosome	151	-0.72	-2.02	0	0
Arachidonic acid metabolism	96	-0.63	-1.71	0	0.007
Lysosome	146	-0.6	-1.67	0	0.0139
**24 weeks**					
Protein digestion and absorption	101	0.64	2.02	0	0.0011
ECM-receptor interaction	89	0.59	1.83	0	0.0099
Oxidative phosphorylation	137	0.55	1.79	0	0.0114
Cardiac muscle contraction	89	0.56	1.72	0	0.0259
Proteasome	48	-0.84	-2.5	0	0
Ribosome biogenesis in eukaryotes	77	-0.57	-1.84	0	0.0102
Basal transcription factors	45	-0.62	-1.81	0.0024	0.0109
RNA transport	175	-0.49	-1.76	0	0.0159
Ribosome	151	-0.49	-1.75	0	0.0145
**36 weeks**					
Butanoate metabolism	28	0.65	1.67	0.0083	0.0477
Signaling pathways regulating pluripotency of stem cells	142	0.49	1.66	0	0.0429
FoxO signaling pathway	134	0.5	1.64	0	0.0434
Circadian rhythm	30	0.63	1.63	0	0.0417
TGF-beta signaling pathway	95	0.51	1.61	0	0.0489
Ribosome	151	-0.78	-2.84	0	0
Lysosome	146	-0.59	-2.12	0	0
Glycolysis / Gluconeogenesis	73	-0.62	-2.02	0	0.0004
Proteasome	48	-0.66	-2.01	0	0.0003
Phagosome	308	-0.46	-1.81	0	0.0068
Amino sugar and nucleotide sugar metabolism	51	-0.55	-1.7	0.0026	0.0145
**60 weeks**					
Oxidative phosphorylation	137	0.68	3.18	0	0
Cardiac muscle contraction	89	0.53	2.23	0	0
Citrate cycle (TCA cycle)	32	0.63	1.99	0	0.0037
Nicotine addiction	40	0.51	1.89	0	0.0022
Retrograde endocannabinoid signaling	154	0.47	1.88	0	0.0018
ECM-receptor interaction	89	0.42	1.72	0	0.0091
Proteasome	48	-0.86	-2.04	0	0
Ribosome biogenesis in eukaryotes	77	-0.65	-1.61	0	0.0019
Spliceosome	150	-0.63	-1.58	0	0.0041
Ribosome	151	-0.61	-1.54	0	0.0066
Basal transcription factors	45	-0.64	-1.5	0	0.0122
Aminoacyl-tRNA biosynthesis	44	-0.61	-1.44	0.0052	0.0302
Protein export	28	-0.63	-1.44	0.0096	0.0314
Protein processing in endoplasmic reticulum	168	-0.57	-1.43	0	0.0312

Table S3 Differentially expressed secreted molecule (predicted), related to Figure 4

Gene id	Symbol	12 weeks		24 weeks		36 weeks		60 weeks	
		FC	FDR	FC	FDR	FC	FDR	FC	FDR
ENSMUSG00000022186	Oxct1	1.39	0	2.04	0	1.78	0	2.36	0
ENSMUSG00000023951	Vegfa	1.55	0.0003	1.48	0.0189	1.54	0	1.3	0.0372
ENSMUSG00000025221	Kcnip2	3.87	0.003	2.96	0	1.47	0.0122	2.04	0
ENSMUSG00000031994	Adamts8	2.16	0	3.8	0	3.75	0	2.67	0.0021
ENSMUSG00000057880	Abat	2.06	0	2.29	0	2.84	0	2.35	0
ENSMUSG00000015568	Lpl	1.66	0	1.21	0.1901	2.16	0	1.51	0
ENSMUSG00000022309	Angpt1	0.84	0.8547	1.7	0.0041	1.95	0	1.52	0.0002
ENSMUSG00000022893	Adamts1	1.89	0.0001	1.6	0.0112	1.38	0.0188	1.03	0.3604
ENSMUSG00000022894	Adamts5	0.92	0.9513	2.19	0	1.33	0.0083	1.26	0.0016
ENSMUSG00000024962	Vegfb	1.23	0.0008	1.28	0.0352	1.03	0.8684	1.61	0
ENSMUSG00000026185	Igfbp5	0.74	0.1516	1.66	0.0112	1.51	0	1.37	0.0086
ENSMUSG00000026399	Cd55	1.24	0.0013	1.96	0	1.2	0.0229	1.03	0.0122
ENSMUSG00000028031	Dkk2	1.37	0.2909	2.29	0.0048	3.74	0	1.68	0.0088
ENSMUSG00000031232	Magt1	1.27	0.0018	1.51	0.0022	1.65	0	0.87	0.7494
ENSMUSG00000031274	Col4a5	1.03	0.9561	2.71	0.0002	2.01	0.0015	1.78	0.0021
ENSMUSG00000037010	Apln	1.13	0.6406	3.23	0	2.38	0.0001	2.71	0
ENSMUSG00000039084	Chad	0.52	0.4885	2.29	0	1.51	0.0023	1.9	0.0492
ENSMUSG00000039496	Cdnf	1.35	0.0001	1.53	0.0019	1.88	0	1.11	0.0989
ENSMUSG00000040972	Igsf21	2.91	0.1348	11.92	0	7.57	0.0024	5.46	0
ENSMUSG00000041193	Pla2g5	0.94	0.9705	2.15	0.0422	2.04	0.0017	1.41	0.0161
ENSMUSG00000043719	Col6a6	0.57	0.0428	2.76	0	1.38	0.0162	1.62	0.0004
ENSMUSG00000048583	Igf2	1.22	0.0114	1.45	0.0285	1.14	0.8493	1.21	0.0001
ENSMUSG00000050821	Fam131a	1.08	0.1716	1.39	0.0168	1.35	0.0001	2.02	0
ENSMUSG00000052276	Ostn	1.4	0.0037	3.82	0	0.87	0.248	2.59	0.0014
ENSMUSG00000079465	Col4a3	0.98	0.9333	2.6	0.0082	1.27	0.0022	2	0.0025

Table S6 Top 15 KEGG enrichment of proteins with differential phosphorylation sites, related to Figure 6

KEGG pathway	Mapping	Background	Fold Enrichment	Fisher's exact test p value
Glycolysis / Gluconeogenesis	13	19	5.75	7.48451E-09
Carbon metabolism	15	30	4.2	1.90919E-07
Biosynthesis of amino acids	10	16	5.25	1.70648E-06
Arrhythmogenic right ventricular cardiomyopathy (ARVC)	12	23	4.38	2.03793E-06
Hypertrophic cardiomyopathy (HCM)	12	26	3.88	1.06135E-05
Dilated cardiomyopathy (DCM)	11	27	3.42	0.000106462
Pentose phosphate pathway	5	6	7	0.000120075
Cardiac muscle contraction	10	24	3.5	0.000180242
Starch and sucrose metabolism	5	7	6	0.000380262
Propanoate metabolism	5	8	5.25	0.000917727
Calcium signaling pathway	13	45	2.43	0.001303551
Glycine, serine and threonine metabolism	3	3	8.4	0.001648541
Galactose metabolism	3	3	8.4	0.001648541
Arginine and proline metabolism	4	6	5.6	0.002370335
Glucagon signaling pathway	10	36	2.33	0.00664018

Table S7 Primers for qPCR, related to Figure S3

Name	Sequence 5'-3'
m18s-f	GTAACCCGTTGAACCCCATT
m18s-r	CCATCCAATCGGTAGTAGCG
Glut1-F	GTGACGATCTGAGCTACGGG
Glut1-R	GAGAGACCAAAGCGTGGTGA
Glut2-F	GCCCAGCAGTTCTCAGGAAT
Glut2-R	ACATGCCAATCATCCCGGTT
Glut3-F	CTTTGGCAGACGCAACTCTAT
Glut3-R	ACCAGAATCCCAACAACGATG
Glut4-F	GGGTGGCCCTGAATTCATCA
Glut4-R	TTCGGGTTTAGCACCCTTCC
Glut5-F	TGCCTTTACCGGGTTGACTC
Glut5-R	GTGTCTTCGCAAATGGCCTG
Glut6-F	AGCAGATCCAGGACAACGTG
Glut6-R	GGCAGCAAACATGACGGATG
Glut10-F	GGGCCTGACCTTCGGATATG
Glut10-R	GCTCCTGTTCGAGGCAACT
Glut12-F	CAGGTACGGAAGAAGGCTCG
Glut12-R	CACACACGTGGCAATGGAAG
